# NIR triggered polydopamine coated cerium dioxide nanozyme for ameliorating acute lung injury via enhanced ROS scavenging

**DOI:** 10.1186/s12951-024-02570-w

**Published:** 2024-06-08

**Authors:** Mingjing Yin, Doudou Lei, Yalan Liu, Tao Qin, Huyang Gao, Wenquan Lv, Qianyue Liu, Lian Qin, Weiqian Jin, Yin Chen, Hao Liang, Bailei Wang, Ming Gao, Jianfeng Zhang, Junyu Lu

**Affiliations:** 1 Intensive Care Unit, The Second Affiliated Hospital of Guangxi Medical University, Nanning, Guangxi 530007 China; 2 Life Sciences Institute, Guangxi Medical University, Nanning, Guangxi 530021 China; 3 Department of Intensive Care Unit, Guangxi Medical University Cancer Hospital, Nanning, Guangxi 530021 China; 4 Department of Emergency, Guangxi Hospital Division of The First Affiliated Hospital, Sun Yat-sen University, Nanning, Guangxi 530022 China; 5 College & Hospital of Stomatology, Guangxi Medical University, Nanning, Guangxi 530021 China

**Keywords:** Acute lung injury, Nanozyme, ROS scavenging, M2 directional polarization, Synergistic enhanced therapy

## Abstract

Acute lung injury (ALI) is a life threatening disease in critically ill patients, and characterized by excessive reactive oxygen species (ROS) and inflammatory factors levels in the lung. Multiple evidences suggest that nanozyme with diversified catalytic capabilities plays a vital role in this fatal lung injury. At present, we developed a novel class of polydopamine (PDA) coated cerium dioxide (CeO_2_) nanozyme (Ce@P) that acts as the potent ROS scavenger for scavenging intracellular ROS and suppressing inflammatory responses against ALI. Herein, we aimed to identify that Ce@P combining with NIR irradiation could further strengthen its ROS scavenging capacity. Specifically, NIR triggered Ce@P exhibited the most potent antioxidant and anti-inflammatory behaviors in lipopolysaccharide (LPS) induced macrophages through decreasing the intracellular ROS levels, down-regulating the levels of TNF-α, IL-1β and IL-6, up-regulating the level of antioxidant cytokine (SOD-2), inducing M2 directional polarization (CD206 up-regulation), and increasing the expression level of HSP70. Besides, we performed intravenous (IV) injection of Ce@P in LPS induced ALI rat model, and found that it significantly accumulated in the lung tissue for 6 h after injection. It was also observed that Ce@P + NIR presented the superior behaviors of decreasing lung inflammation, alleviating diffuse alveolar damage, as well as promoting lung tissue repair. All in all, it has developed the strategy of using Ce@P combining with NIR irradiation for the synergistic enhanced treatment of ALI, which can serve as a promising therapeutic strategy for the clinical treatment of ROS derived diseases as well.

**Supplementary Information:**

The online version contains supplementary material available at 10.1186/s12951-024-02570-w.

## Introduction

Acute lung injury (ALI) is a clinical syndrome associated with extensive pneumonia, severe hypoxemia, and respiratory failure [1]. And, in some cases, it could lead to a more serious form of acute respiratory distress syndrome with a mortality ratio of 25%~40%. Recently, the number of patients with ALI, caused by direct or indirect injuries like infections, severe trauma, aspiration of gastric contents, hemorrhagic shock, and sepsis to lung tissue, has dramatically increased [2]. As it often results in incomplete repair caused by severe injury to alveolar epithelium and lung endothelial cells, it significantly affects the long-term quality of life for some survivors [3]. Therefore, ALI therapy has received increased basic and clinical research interests.

Many studies on diseases mechanism indicate that the progression of ALI is often characterized by excessive reactive oxygen species (ROS) generation causing oxidative injury in the lung throughout the entire circulatory system [4]. The over-produced ROS can activate the inflammatory response, causing pulmonary infiltration of immune cells, like neutrophils and macrophages, and excessive secretion of inflammatory cytokines [5, 6]. On the other hand, inflammatory reactions can lead to the change of intracellular and extracellular environments, thereby increasing the production of ROS, and causing oxidative stress [7, 8]. Thus, it indicates that scavenging ROS is one of the important pathways for antioxidant and anti-inflammation, and control of excessive ROS is crucial for the treatment of ALI.

Nanozymes are a series of nanosized catalysts with multiple enzyme mimicking behaviors [9–11]. They are recognized as promising nanobiomedicine alternatives widely applied in biomedical fields due to good biocompatibility, enzymatic activity, relative stability, and low cost [12–14]. Among them, CeO_2_ nanoparticles (NPs) have received considerable attentions as nanozymes for diseases therapy owing to their low biotoxicity, excellent biocompatibility, and multiple catalytic activity [15]. Due to their tunable valence state transition (Ce^4+^/Ce^3+^) and oxygen vacancy, CeO_2_ possesses similar catalytic activities to that of other NPs [16]. However, the previously reported catalytic activities of CeO_2_ are quite poor, which greatly limits their further applications in catalytic therapy. Recent CeO_2_ based nanosystems for diseases therapy mainly rely on forming combined nanoplatforms by conjugating them with targeted ligands [17], photosensitizers [18] and therapeutic drugs [19]. Promisingly, not just as drug carriers, CeO_2_ could act as hybrid enzymes-like therapeutic agents, such as converting ·O_2_^−^ to O_2_ [20], or inducing ROS formation with external stimuli [21]. To further strengthen its catalytic activities, CeO_2_ was also engineered designed to generate rich defects by doping different metal or metal oxide including Au [16], Pt [22], Ru [23], Cu [24], Mn [25] etc., or different polymer materials (polyvinylpyrrolidone (PVP) [26], polymethyl methacrylate (PMMA) [27], polyaniline (PANI) [28] etc.). However, the current doped CeO_2_ based nanozymes still need to be assisted by photothermal effect [29], ultrasonic driven [30], or UV stimuli [31] to boost their general properties, thus achieving the optimal therapeutic effects with low dosage of nanozymes. Meanwhile, polydopamine (PDA) and its derivatives have been considered as efficient strategies for diseases therapy owing to their excellent biocompatibility, near infrared (NIR) absorbance, and ROS scavenging capacity [32]. Significantly, by hybridizing PDA with metal or metal oxide like Pt [33], Fe_3_O_4_ [34], Mn_3_O_4_ [35], etc., it displayed the enhanced ROS scavenging and inflammation elimination capacities together with improved dispersion and stability [36], further leading to synergistic therapeutic effects of various diseases [37].

**Fig. 1:**
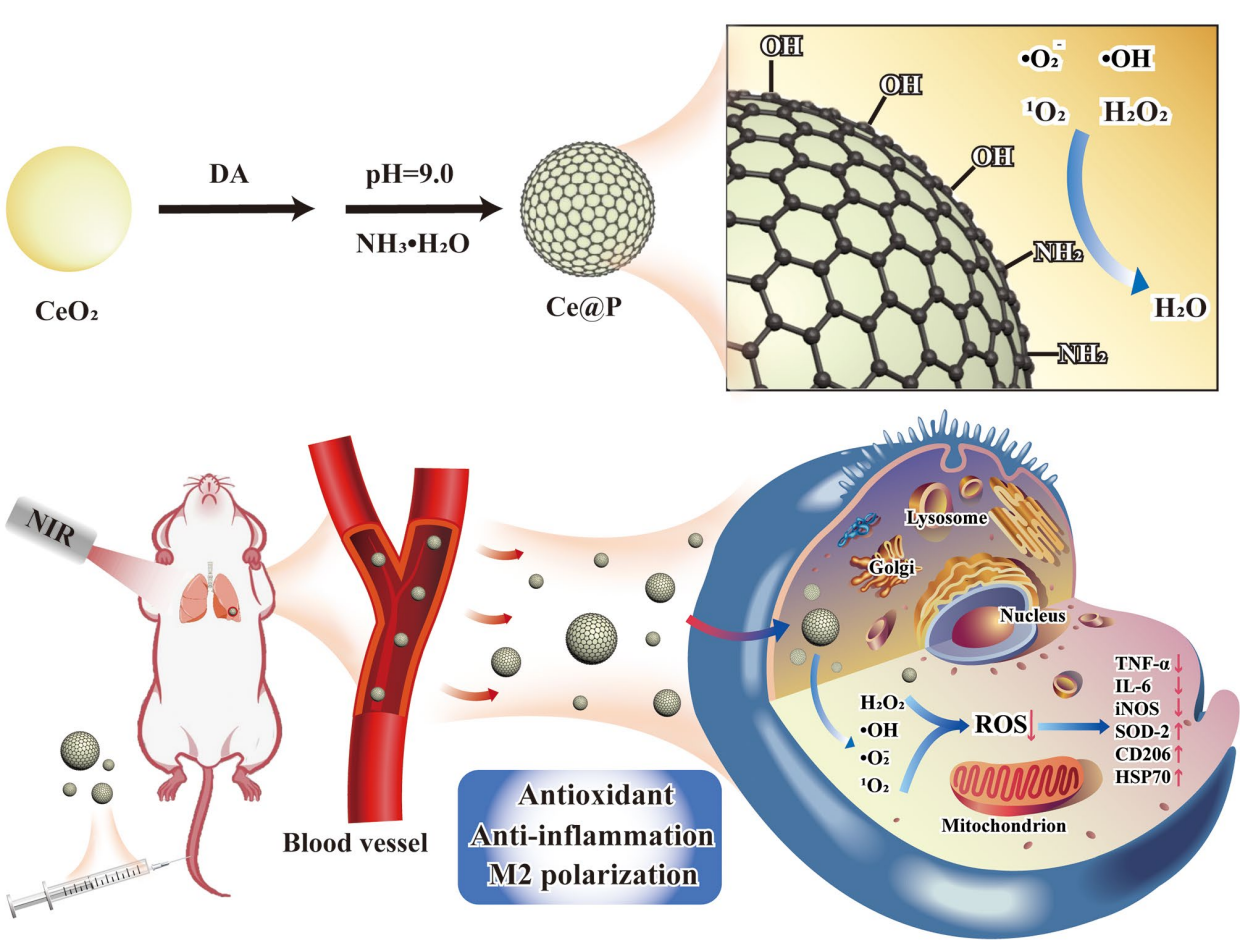
Schematic illustration of synthesis of Ce@P and in vivo ALI therapy by intravenous (IV) injection of Ce@P. The synergistic enhanced ALI therapy was achieved by the strategy of Ce@P combining with NIR irradiation via antioxidant, anti-inflammation and M2 directional polarization

Inspired by the excellent catalytic activities of nanozymes, we designed the novel nanozyme (Ce@P) through encapsulating CeO_2_ with PDA, combining with NIR irradiation, to activate anti-inflammation pathway and M2 polarization pathway, further to regulate the intracellular redox homeostasis for ameliorating ALI (Fig. 1). Simultaneously, CeO_2_ had multiple catalytic activities, standing out as a biocompatible core. After coating with PDA, Ce@P exhibited enhanced ROS scavenging and photothermal activities compared to CeO_2_ alone, contributing to the synergistic enhanced effect of ALI therapy. As revealed by LPS induced macrophages and ALI rat models, it exhibited favorable biosafety, and efficient antioxidant and anti-inflammation capacities, significantly contributing to the alleviation of ALI. Summarily, it could be promoted as an efficient strategy for the therapy of various ROS type diseases.

## Materials and methods

### Reagents and chemicals

3-hydroxytyramine hydrochloride (DA, ≥ 98%), ammonium hydroxide (NH_4_OH, 25.0 ~ 28.0%), ethanol (≥ 99%) and cerium dioxide (CeO_2,_ 95%) were purchased from Aladdin (China). Lipopolysaccharide (LPS) was commercially obtained from Sigma-Aldrich (St. Louis, MO) and hydrogen peroxide (H_2_O_2_) (30% w/w) were supplied from Junobio (Nanning, China). All reagents were directly used without any treatments.

### Preparation and characterization of Ce@P

The preparation of Ce@P was implemented by a simple method as previously reported [38]. The details were as following: 1 g CeO_2_ was fully dispersed in 100 mL deionized (DI) water. After total dispersion by magnetic stirring, 0.2 g DA was dissolved in 10 mL DI water and then added into the mixture followed by adjusting pH = 9.0. And the mixture reacted overnight before purification by repeated dispersion and centrifuge for 3 times (Table S1). The final product was obtained by vacuum drying and kept for the following experiments.

To investigate the crystallization structure, molecular structure, element composition and metal element contents of NPs, X-ray diffraction (XRD, MiniFlex 900, Japan), Fourier transform infrared spectrometer (FTIR, Shimadzu, Japan), X-ray photoelectron spectroscopy (XPS, ESCALAB 250XI+, USA) and inductively coupled plasma mass spectrometer (ICP-MS, Thermo, USA) were applied respectively. And the morphology and elemental distribution of Ce@P were analyzed by transmission electron microscopy (TEM) coupled with energy-dispersive X-ray spectroscopy (EDS) (Hitachi, Japan) respectively. In the meantime, the zeta potential was tested by zeta sizer (Nano ZS90, Malvern, UK). In addition, to investigate its dispersion ability and stability, Ce@P was respectively dispersed in PBS, Dulbecco’s modified eagle medium (DMEM, Solarbio, China), fetal bovine serum (FBS, Solarbio, China), or 5 mM H_2_O_2_, followed by being observed by camera at different time points. Furthermore, thermal gravimetric analysis (TGA, STD650, TA, USA) was applied to investigate the thermal stability of Ce@P.

### Photothermal behaviors testing

The investigation of photothermal properties was respectively implemented by placing NPs with various concentrations of 0, 50, 100–200 µg/mL in PBS buffer under NIR irradiation (808 nm, 0.5, 1, 1.5–2 W/cm^2^) for a certain of time. Particularly, the photothermal stability was investigated by preparing Ce@P with 100 µg/mL under NIR irradiation of 4 repeated “on” and “off” cycles. The corresponding images and temperatures were recorded and saved by thermal imaging camera (FLIR, USA) during NIR irradiation.

### ROS scavenging capacity investigation

ROS scavenging capability of NPs was initially implemented by utilizing ROS (H_2_O_2_, ·OH, and ·O_2_^−^) testing kits (Solarbio, China) by following the protocols. Briefly, for H_2_O_2_ scavenging ability, different concentrations of NPs (50, 100 and 200 µg/mL) were dispersed in the corresponding working solutions. And the absorbance was observed at 520 nm by a microplate reader (Thermo Scientific, USA) after reacting for 10 min. Similarly, the absorbance was respectively obtained at 536 nm and 450 nm by using the microplate reader for investigating ·OH and ·O_2_^−^ scavenging capacity. Specifically, for testing ROS scavenging ability of Ce@P + NIR, NIR irradiation was implemented for 10 min (808 nm, 1.5 W/cm^2^) during reaction followed by the observation of microplate reader. Additionally, ROS scavenging capability was also investigated by electron spin resonance (ESR, Bruker A300, Germany). Briefly, 5-tert-butoxycarbonyl 5-methyl-1-pyrroline-N-oxide (BMPO, 100 mM), xanthione (10 mM) and xanthione oxidase (XOD, 1U/mL), and 2, 2, 6, 6-Tetramethylpiperidine (TEMPONE, 100 mM) were applied as the working solution for ·OH, ·O_2_^−^ and ^1^O_2_ testing respectively. After mixing the working solutions with 100 µg/mL NPs for 10 min, the signal of solutions was recorded by ESR. Specifically, for Ce@P + NIR, NIR irradiation (808 nm, 1.5 W/cm^2^) was implemented for 10 min before ESR testing.

### Cell biocompatibility and cellular uptake evaluation

The murine macrophage cells (RAW264.7) were commercially obtained from American type culture collection (ATCC, USA) before being cultured in DMEM containing FBS (10%) and penicillin/streptomycin (1%, Procell, China). And the cells were passaged after reaching 90% confluence, and the third passage was applied for the following experiments.

The cell biocompatibility was investigated by using cell counting kit-8 (CCK-8, Biosharp, China) following the procedures of manufacture. The detailed steps were: cells were cultured in the microplate with the density of 5000 cells/well, and replaced with CeO_2_ or Ce@P solutions in various concentrations of 0, 5, 10, 20, 50, 100, 200 and 500 µg/mL. After incubation for 24 h, the cultured medium was added with CCK-8 solution (100 µL, 1 h) after washing against with PBS buffer. And the corresponding absorbance was recorded at 450 nm by spectrophotometer (Thermo Fisher, USA).

In addition, the protection capacity was implemented by live/dead staining of cultured cells. In brief, cells were induced with LPS (1 µg/mL) for 30 min followed by incubating with different NPs (100 µg/mL) for 24 h. Specifically, for Ce@P + NIR, NIR irradiation was implemented for 3 times (5 min per time, 1.5 W/cm^2^) at 0, 1 and 2 h after incubation with NPs. Next, the cells were treated with calcein-AM/propidium iodide (PI) (Beyotime Biotechnology, China) for 5 min in darkness, and imaged by fluorescent microscopy (Olympus, Japan) after rinsing with PBS buffer for 3 times. Meanwhile, live/dead staining was also applied to test the viability of cells after Ce@P incubation under NIR irradiation (808 nm, 1.5 W/cm^2^) for 0, 5, 10 and 15 min.

To investigate hemocompatibility, hemolysis test was implemented. The fresh blood was collected from Sprague-dawley (SD) rats, and saved in heparin treated blood collection vessels. After centrifuging for 15 min at 3000 rpm, erythrocyte pellet was collected after removing the supernatant. And the erythrocyte pellet was re-suspended in PBS buffer. Next, 0.5 mL erythrocyte suspension was mixed with 0.5 mL Ce@P solutions in various concentrations (0, 5, 10, 20, 50 100, 200 and 500 µg/mL), where 0.5 mL DI water was used as positive control, and 0.5 mL PBS was used as negative control. After incubating for 1 h, the mixture was centrifuged at 3000 rpm for 15 min, and 100 µL supernatant was observed by microplate reader at 540 nm. And the hemolysis ratio was calculated as following: hemolysis ratio (HR) =[(OD_s_-OD_n_)/(OD_p_-OD_n_)]/100, where OD_n_, OD_p_ and OD_s_ was the optical density (OD) of negative control, positive control and each samples respectively.

Finally, the cellular uptake capacity was investigated as follows: Firstly, Cy5 labeled NPs were prepared by mixing 50 mg CeO_2_ or Ce@P (0.5 mg/mL in DMSO) with 0.5 µg/mL Cy5-PEG2000-Thiol (Lumiprobe, China) for 24 h, and collected for further experiments by vacuum drying after repeated washing with methanol for 3 times. On the other hand, cells were cultured in confocal dishes and incubated with Cy5-CeO_2_ or Cy5-Ce@P for 3 h. Later, cells were washed by PBS buffer before fixing with 4% paraformaldehyde (PFA, Biosharp, China). Meanwhile, the cytoskeleton of cells was also stained by actin-tracker green-488 (actin, Biosharp, China) (30 min), and the nuclei was stained with 4, 6-diamidino-2-phenyindole dilactate (DAPI, Biosharp, China) (10 min) by following the protocols. After staining, cells were washing against with PBS for 3 times. Finally, after embedding in paraffin, the images were photographed by confocal scanning microscope (ZEISS, Germany).

### Antioxidant and anti-inflammatory investigation in cellular level

The intracellular ROS levels testing of cells were implemented by utilizing ROS testing kits (Beyotime, China) following the protocols of manufacture. In brief, LPS (1 µg/mL, 30 min) induced macrophages were treated with 100 µg/mL NPs overnight. Next, the medium was replaced with fresh staining mixtures containing 2’, 7’-dichlorofluorescin diacetate (DCFA, maokangbio, China) for total ROS testing, dihydroethidium (DHE, maokangbio, China) for ·O_2_^−^ testing, and hydroxyphenyl fluorescein (HPF, maokangbio, China) for ·OH testing respectively by following the protocols before being imaged by fluorescent microscopy.

Besides, the levels of inflammatory factors were initially identified by enzyme-linked immunosorbent assay (ELISA). After incubation with NPs for 24 h, the supernatant of cells was collected, and detected by the corresponding ELISA kits (Meimian, China) after following the instructions.

Furthermore, the inflammatory and anti-inflammatory levels were verified by immunofluorescence staining. In details, the treated macrophages were fixed by 95% methanol for 1 h before being blocked with goat serum, and incubated with primary antibody: IL-6, CD206, iNOS or HSP70 (1:200 dilutions, Proteintech, China) respectively at 4℃ overnight. After adding secondary antibody (FITC-anti-rabbit IgG (Boster, China)) for another 1 h, and staining the nuclei with DAPI for 10 min, the images were obtained by fluorescent microscope.

Finally, the inflammatory genes (TNF-α), M1 type genes (IL-6 and iNOS), anti-inflammatory genes (SOD2), M2 type gene (CD206), and heat shock protein gene (HSP70) expression levels were analyzed by quantitative real-time PCR (qRT-PCR). Briefly, after treatment, total RNA from macrophages was extracted by RNA extraction kit (Vazyme, China) by following the protocols, and qRT-PCR was implemented by Real-Time PCR System (Aglien, USA). And the relative genes levels were analyzed by 2^−ΔΔCt^ method and compared with β-actin (ACTB). In Table S2, the corresponding primer sequences were listed.

### In vivo bio-distribution, photothermal and biosafety investigation

In vivo experiment was conducted with the approval of ethics committee of animal experiments of Guangxi Medical University. All animals were taken good care according to the local guide for the care and use of laboratory animals of Guangxi Medical University. SD rats (180 ~ 220 g) were housed under standard specific pathogen-free (SPF) grades, and given free drinking and safe eating with a controlled indoor environment.

To evaluate in vivo distribution of Ce@P, in vivo animal imaging systems (IVIS, Pekin Elmer, US) were applied. In details, SD rats were intravenous (IV) injected with 0.8 mL sample solutions (100 µg/mL Ce@P, Cy5 or Cy5-Ce@P) respectively. At predetermined time points (0, 0.5, 1, 2, 6 and 24 h), major organs were isolated, and imaged by IVIS with excitation wavelength at 646 nm and emission wavelength at 664 nm respectively.

In vivo photothermal effects were evaluated by the following procedures. Details were: 0.8 mL Ce@P (100 µg/mL, 5 mg/kg) was IV injected into SD rats. After 1 h, the lung of rats was irradiated by NIR light (1.5 W/cm^2^) for 15 min, and the corresponding images were collected every min by thermal camera.

For in vivo biosafety evaluation, SD rats were IV injected with 0.8 mL PBS or Ce@P (100 µg/mL) respectively. The body weight of each rats was tested daily for 7 day. And the blood samples of rats at 7 day were analyzed by blood routine test (fully automatic blood analyzer, BC-6800Plus, Mindray, China), blood biochemistry test (fully automatic biochemical analyzer, 7600, HITACHI, China), and coagulation function test (fully automatic coagulation detection analyzer, ACL TOP750, Werfen, China) respectively. Besides, the major organs such as heart, liver, spleen, lung and kidney were isolated, sectioned and stained by hematoxylin and eosin (H&E) for the observation of Olympus microscope.

### In vivo therapy evaluation

ALI animal model was established by dripped infusion of SD rats with LPS (1 mg/mL, 5 mg/kg) into the trachea after cutting the skin (1 cm length) along the midline of neck, and exposing the trachea. After suturing the skin, SD rats were saved for further experiments. SD rats were divided into 4 groups: sham group (rats without operations), ALI group (ALI rats with 0.8 mL PBS injection), Ce@P (ALI rats with Ce@P injection (0.8 mL, 100 µg/mL)), and Ce@P + NIR (ALI rats with Ce@P injection (0.8 mL, 100 µg/mL) and NIR irradiation (1.5 W/cm^2^)). SD rats were IV injected with the above mixtures after 2 h’ modeling and sacrificed at 24 h after treatment. Specifically, for Ce@P + NIR, NIR irradiation was implemented for 3 times, 5 min per time respectively at 1, 2 and 3 h after IV injection of Ce@P. And the major organs and blood samples were saved for further evaluations. The blood samples of each group were assessed by blood routine test, blood biochemistry test, and coagulation function test respectively.

ALI therapy effects mainly focused on the research of lung tissue. After taking out from rats, the lungs were imaged, and their wet/dry ratios were also calculated by weighing the lungs before and after oven drying. Besides, the inflammatory factors levels of lung tissue were identified by ELISA. The lung tissues were cut and homogenized before centrifuging at 3000 rpm for 10 min. And the total lung homogenates were prepared to detect TNF-α, IL-6 and IL-1β levels by ELISA. And the ROS levels of lung sections were analyzed by using ROS probe (DHE) followed by DAPI staining. After staining and mounting, the slides were observed by fluorescent microscope. Additionally, the superoxide dismutase (SOD) activity of lung homogenates was investigated by total SOD activity detection kit (Beyotime, China) following the instruction of manufacture. And the lipid peroxidation levels were also detected by malondialdehyde (MDA) detection kit (Solarbio, China).

Furthermore, the lungs were fixed by 4% PFA overnight, and cut into 3 μm thickness for H&E staining. At last, the slides were observed by Olympus microscopy and evaluated by Smith scoring. In particular, for immunohistochemical staining, the section slides were incubated with first antibody (rabbit polyclonal anti-HSP70, Servicebio, China) at 4 °C overnight followed by binding with biotinylated secondary antibodies (Servicebio, China). After sealing, the slides were imaged by Olympus microscope. Meanwhile, the other organs (heart, liver, spleen and kidney) from rats were also fixed by PFA and cut into 3 μm thickness for H&E staining.

### Statistical analysis

All experiments were implemented at least in triplicates. And the data were presented as mean ± standard deviation. Unless otherwise stated, the statistical significance of results was analyzed using one-way ANOVA followed by least significant difference (LSD) analysis.

## Results and discussion

### Preparation and physicochemical properties

**Fig. 2:**
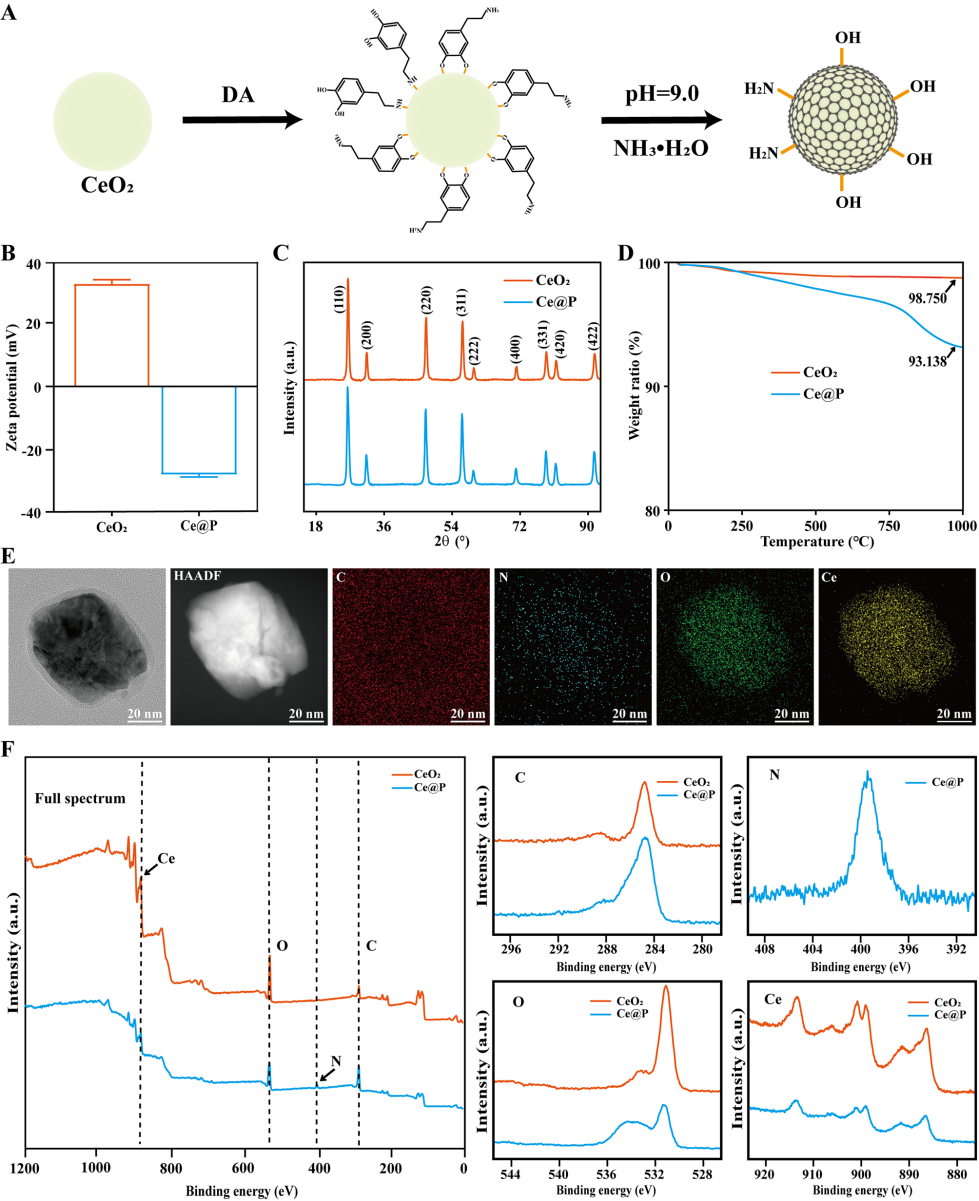
Preparation and basic characterization of NPs. **A**) Synthesis procedure of Ce@P. **B**) Zeta potential of CeO_2_ and Ce@P. **C**) XRD results of CeO_2_ and Ce@P. **D**) TGA results of CeO_2_ and Ce@P. **E**) TEM-mapping results of Ce@P and the corresponding element composition (HAADF, C, N, O and Ce images). **F**) XPS results of CeO_2_ and Ce@P (Full spectrum, C, N, O and Ce detailed spectrum)

Nanosized CeO_2_ was commercially purchased without further purification. And Ce@P was prepared by dispersing CeO_2_ in DI water followed by adding DA solution. The whole reaction kept at pH = 9. The polyphenol groups of DA were adsorbed on the surface of CeO_2_ and the redox polymerization occurred under alkaline condition. After encapsulating CeO_2_ with PDA layers, it formed Ce@P (Fig. 2A). As shown in Fig. S1, it was yellow for CeO_2_ and became black for Ce@P after PDA coating. And the zeta potential of NPs was also analyzed. As illustrated in Fig. 2B and Table S1, the zeta potential was 33.17 ± 1.38 mV for CeO_2_, changing to -28.50 ± 0.79 mV for Ce@P. Thus, encapsulating with PDA could change the color and zeta potential of Ce@P. It was well known that XRD was always applied to evaluate the crystallization change of NPs. Thus, the crystallization structure of NPs was further characterized by XRD. As shown in Fig. 2C, it was obviously observed that some characteristic peaks appeared for CeO_2_, consistent with the reported results [39]. However, compared with CeO_2_, it maintained almost the same crystallinity for Ce@P, demonstrating that PDA coating did not change the crystallization structure in this case. Meanwhile, the molecular structure of NPs was also characterized by FTIR. As illustrated in Fig. S2, the obvious characteristic peak were observed around 3230 cm^− 1^ and 1610 cm^− 1^ for PDA, corresponding to its hydroxyl group (-OH) and carbonyl group (C = O). However, obvious peak at 1630 cm^− 1^ existed for CeO_2_, attributed by H_2_O peak during testing. Compared to CeO_2_, slight peaks at 3230 cm^− 1^ and 1610 cm^− 1^ appeared for Ce@P contributed by PDA coating. Thus, PDA coating slightly changed the molecular structure of Ce@P.

Besides, it displayed almost the same thermal properties with only slight differences in weight loss ratio, which was 1.25% for CeO_2_ and 6.86% for Ce@P respectively (Fig. 2D). By calculation, it was 5.61% PDA coating for Ce@P. Moreover, the morphology of NPs was characterized by TEM-mapping. No obvious differences were observed between the morphology of CeO_2_ and Ce@P, both around 60 nm in square shape (Fig. 2E and S3). And it indicated that only Ce and O elements existed for CeO_2_ while C, N, O and Ce elements appeared for Ce@P (Fig. 2E and Fig. S3). The extra occurrence of C and N elements was attributed to PDA coating for Ce@P. In the meantime, the element composition of NPs was also analyzed by XPS. Only Ce and O elements were observed for CeO_2_ while C and N elements were emerged for Ce@P (Fig. 2F), indicating the proof of PDA coating on Ce@P as well. The extra C element observed for CeO_2_ was attributed to the carbon film during testing. And the weight ratio was 58.67%, 6.65%, 30.44% and 4.23% for C, N, O and Ce respectively for Ce@P (Table S3). Specifically, the content of Ce element was 32.20 ± 0.29% for Ce@P by ICP-MS (Table S3).

**Fig. 3:**
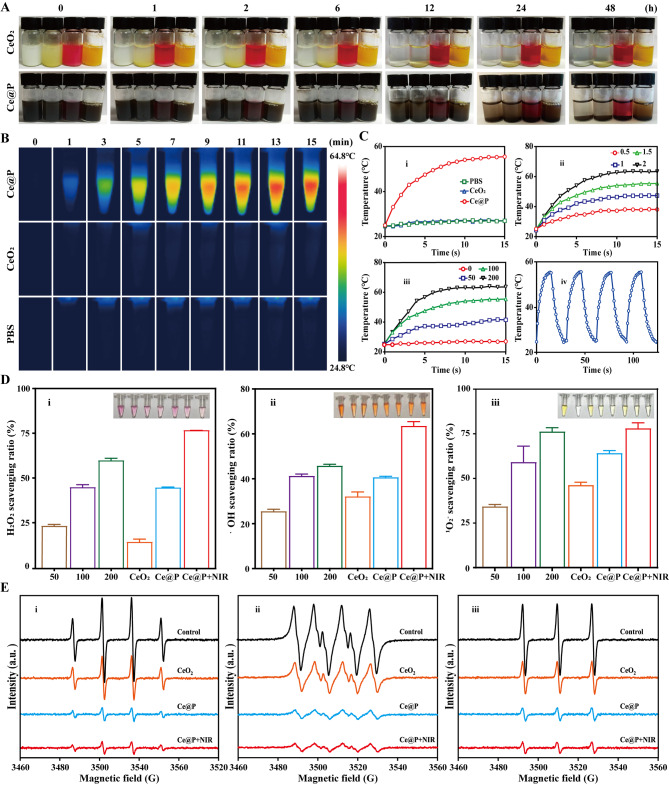
Physicochemical properties of NPs. **A**) Dispersion and stability of CeO_2_ and Ce@P at predetermined time points (0, 1, 2, 6, 12, 24 and 48 h), and the corresponding solutions were PBS, H_2_O_2_ (5 mM), DMEM and FBS respectively (from left to right). **B**) Photothermal images of different NPs (CeO_2_ and Ce@P) with the same concentration of 100 µg/mL under NIR irradiation (1.5 W/cm^2^) versus time. **C**) Temperature changes of PBS, CeO_2_ and Ce@P with the same concentration of 100 µg/mL under NIR irradiation (1.5 W/cm^2^) (i), 100 µg/mL Ce@P under different power intensity of NIR irradiation (0.5, 1, 1.5 and 2 W/cm^2^) (ii), different concentrations (0, 50, 100 and 200 µg/mL) of Ce@P under NIR irradiation (1.5 W/cm^2^) (iii) versus time, and photothermal stability of 100 µg/mL Ce@P under NIR irradiation (1.5 W/cm^2^) for 4 “on” and “off” cycles (iv). **D**) ROS scavenging capacity of different NPs by ROS testing kits: H_2_O_2_ (i), ·OH (ii) and ·O_2_^−^ (iii). E) ESR results of ROS scavenging ability of different NPs: ·OH (i), ·O_2_^−^ (ii) and ^1^O_2_ (iii)

Furthermore, the in vitro dispersion and stability of NPs were studied by dispersing CeO_2_ and Ce@P in PBS, 5 mM H_2_O_2_, DMEM and FBS respectively, and observed at predetermined time points (0, 1, 2, 6, 12, 24, and 48 h). From Fig. 3A, it displayed the actual dispersion conditions of CeO_2_ and Ce@P versus time. CeO_2_ was well dispersed at the beginning in PBS and begun to deposit after 2 h, proved by the transparent supernatant on the top and obvious CeO_2_ on the bottom. It was the similar condition for CeO_2_ in DMEM, FBS and H_2_O_2_. Specifically, it was observed that CeO_2_ could be totally dissolved in H_2_O_2_ with turbid solution observed. However, it presented relative stable dispersion for Ce@P. At 12 h, it was still dispersed in 4 different solutions, with the turbid solutions observed. And the supernatants became transparent for PBS, H_2_O_2_, DMEM and FBS at 48 h. Thus, compared to CeO_2_ alone, Ce@P presented a certain of stability in 4 different solutions due to the fact that PDA coating could shield the interaction between NPs and also protect CeO_2_ avoiding from quick degradation [40].

As a non-invasive treatment method, NIR irradiation was considered to achieve the photothermal therapy of various diseases [41]. And the photothermal properties of NPs were investigated by monitoring the temperature changes of different concentrations of NPs under NIR irradiation with different irradiation intensity. As shown in Fig. 3B, there was nearly no temperature changes for CeO_2_ under NIR irradiation for 15 min, almost close to those of PBS maintaining the temperature range from 24.80℃∼27.00℃. Nevertheless, with the same concentration, obvious changes of temperature were observed for Ce@P, which could increase to 55.50℃ under irradiation while it was only 27.00℃ and 27.10℃ for PBS and CeO_2_ respectively (i of Fig. 3B). PDA was a well-known kind of photothermal agents [32]. The yellow CeO_2_ went against NIR adsorption, further reducing photothermal effect. And the photothermal effect had apparently been improved after PDA coating for Ce@P, possible to collect more NIR. Thus, Ce@P possessed excellent photothermal effects after PDA coating. In addition, for 100 µg/mL Ce@P, if increasing NIR irradiation intensity, the corresponding temperature also increased (ii of Fig. 3B). Meanwhile, if increasing Ce@P concentrations from 0, 50, 100 to 200 µg/mL, the corresponding temperatures separately jumped from 27.00 to 63.60℃ (iii of Fig. 3C). Under the same irradiation intensity, increasing the concentration of Ce@P was equal to high photothermal conversion. Significantly, after 4 “on” and “off” cycles, the heating and cooling processes still remained consistent, displaying the stable photothermal conversion (iv of Fig. 3C).

Finally, ROS scavenging capacity was respectively evaluated by using ROS testing kits and ESR. As illustrated in Fig. 3D, it apparently displayed H_2_O_2_ (i), ·OH (ii) and·O_2_^−^ (iii) clearance ability by NPs. CeO_2_ showed 14.27 ± 1.60% scavenging ratio of H_2_O_2_ and it became 44.63 ± 0.33% for Ce@P. By combining with NIR irradiation, the scavenging ratio increased to 76.70 ± 0.12% (i of Fig. 3D and Table S4). Meanwhile, increasing the dosage concentration of Ce@P from 50, 100 to 200 µg/mL, the corresponding H_2_O_2_ scavenging ratio also jumped from 23.21 ± 0.85%, 44.84 ± 1.46% to 59.82 ± 1.22% (Table S5), indicating that scavenging capacity was also concentration dependent. It presented the similar tendency for ·OH and ·O_2_^−^ scavenging, with the scavenging order of Ce@P + NIR>Ce@P>CeO_2_. It was 32.12 ± 2.02% ·OH scavenging ratio and 46.21 ± 1.64% ·O_2_^−^ scavenging ratio for CeO_2_, increased to 40.58 ± 0.51% and 64.11 ± 1.50% for Ce@P, and 63.50 ± 1.92% and 78.05 ± 3.11% for Ce@P + NIR respectively with the dosage concentration of 100 µg/mL (ii and iii of Fig. 3D and Table S4). Additionally, ROS scavenging capacity was evaluated by ESR. The intensity of ESR corresponded to ROS level. As displayed in i of Fig. 3E, it was observed that the intensity of control group was in high levels for ·OH, which became weaken after the dosage of NPs, indicating the occurrence of ROS scavenging. CeO_2_ could slightly decrease the intensity of ESR, enhanced by Ce@P and Ce@P + NIR. Similarly, it displayed the same tendency for ·O_2_^−^ and ^1^O_2_ scavenging (ii and iii of Fig. 3E). However, no significant differences were observed between Ce@P and Ce@P + NIR by ESR, possibly due to the fact that it only provided qualitative results of ROS scavenging and could not truly reflect the quantitative results by ESR. Although only slight differences existed between Ce@P and Ce@P + NIR by ESR, the overall trends still remained consistent with those of ROS testing kits. PDA NPs had confirmed their ROS scavenging capacities due to their huge amount of phenolic hydroxyl groups [42]. Thus, Ce@P possessed better ROS scavenging capacities than those of CeO_2_, indicating that PDA coating contributed the enhanced scavenging effects, consistent with the previously reported results [43]. On the other hand, NIR irradiation was helpful to the movement of Ce@P, leading to the improvement of ROS scavenging [44].

From the above results, it gave the proof of the successful preparation of Ce@P. After PDA coating, it could not significantly change the crystallization structure, molecular structure and morphology structure, but affected the zeta potential and element composition. And PDA coating was helpful to improve the dispersion and thermal stability. Significantly, PDA coating contributed to enhanced photothermal behaviors and ROS scavenging capacity, showing promising potentials in clinic application.

### Biological functions in cellular levels

#### Cell biocompatibility and protection ability

**Fig. 4:**
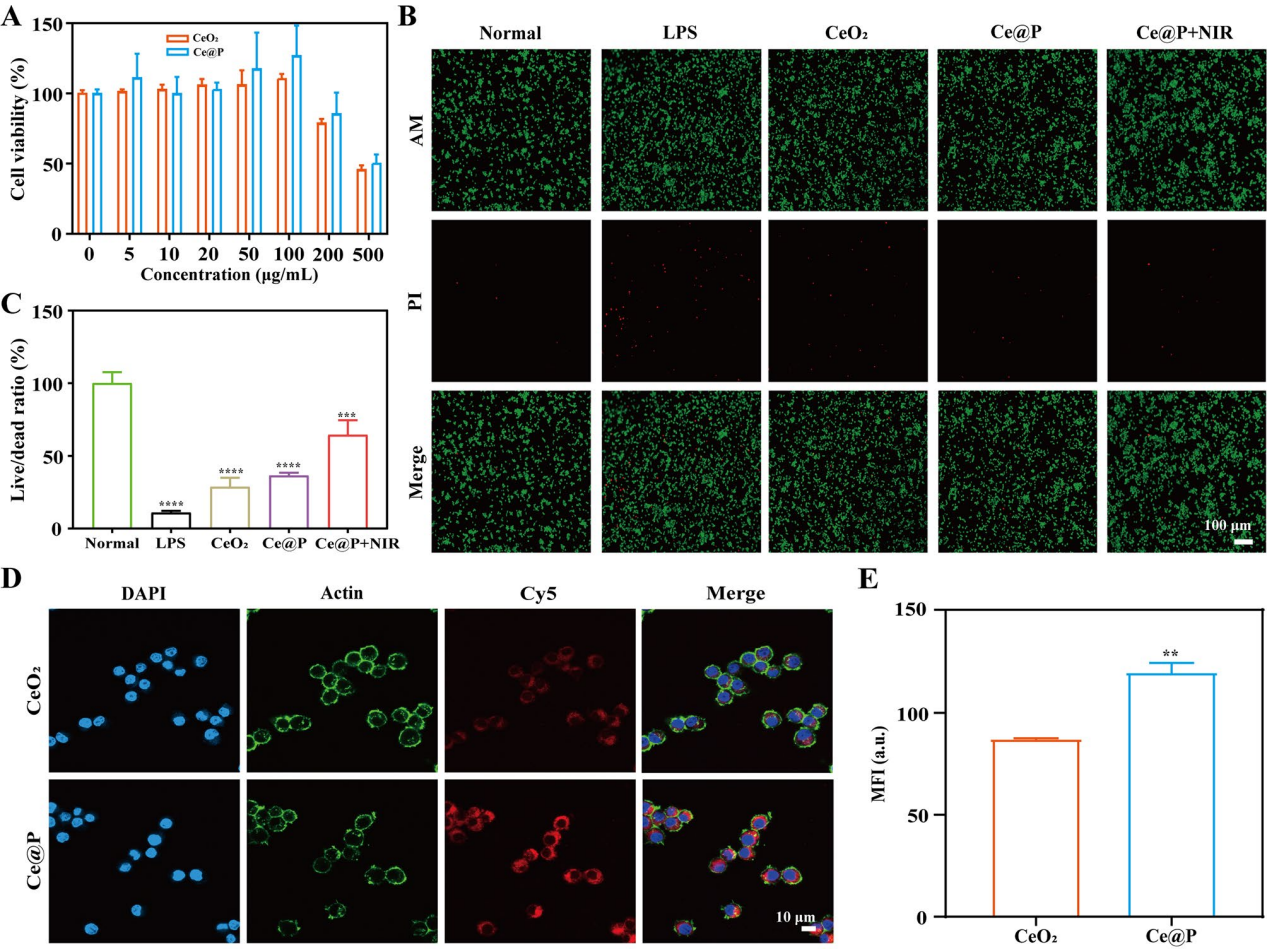
Cell biocompability and cellular uptake capacity. **A**) Cell viability of CeO_2_ and Ce@P with different concentrations ranging from 0 to 500 µg/mL. **B**) Live/dead staining images of treated cells and their corresponding quantified results (**C**). The corresponding groups were: cells without treatment (normal group), cells pre-treated with LPS followed by incubating with PBS buffer (LPS group), cells pre-treated with LPS followed by incubating with 100 µg/mL CeO_2_ (CeO_2_), cells pre-treated with LPS followed by incubating with 100 µg/mL Ce@P (Ce@P), and cells pre-treated with LPS followed by incubating with 100 µg/mL Ce@P and NIR irradiation (1.5 W/cm^2^) (Ce@P + NIR). **D**) Cellular uptake images of cells incubated with Cy5-CeO_2_ and Cy5-Ce@P for 3 h by confocal microscope and the corresponding quantified results (E). (“*” symbol compared with CeO_2_, **p < 0.01)

Cell cytotoxicity was investigated by incubating cells with different concentrations of NPs followed by CCK-8 assay. From Fig. 4A, it was observed that both CeO_2_ and Ce@P possessed good biocompability in the concentrations ranging from 0 to 100 µg/mL, with cell viability above 95%. However, Ce@P presented a certain of cytotoxicity if increasing its concentration to 200 µg/mL. Thus, 100 µg/mL was considered to be applied for further experiments.

LPS intervention was commonly applied to establish in vitro and in vivo ALI models, due to the fact that it was similar to the pathological characteristics of ALI patients [45–47]. Thus, the protection ability was evaluated by live/dead staining of LPS stimulated cells followed by NPs incubation. As shown in Fig. 4B, LPS induced a large amount of dead cells (red fluorescence) with the live/dead ratio of 10.79 ± 1.39% while a lot of live cells (green fluorescence) and few dead cells were observed for normal group with 100.00 ± 7.73% live/dead ratio. And it was also found that CeO_2_ could slightly improve the live/dead ratio to 28.60 ± 6.33% with a certain of green fluorescence. In particular, Ce@P obviously improved the protection ability of LPS induced cells with live/dead ratio of 36.30 ± 2.09%. Most significantly, with NIR irradiation, the live/dead ratio increased to 64.30 ± 10.30% with only few dead cells observed (Fig. 4C). The above results revealed that LPS induced oxidative stress resulted in the death of cells. Compared to CeO_2_, Ce@P with enhanced ROS scavenging capacity could more efficiently prevented LPS induced cell death. Specifically, the ability of preventing LPS induced cell death could be enhanced by photothermal effects. In addition, cell viability under NIR irradiation (1.5 W/cm^2^) was evaluated by live/dead staining. As displayed in Fig. S4, few dead cells were observed for Ce@P + NIR under different NIR irradiation time. By statistical calculation, the live/dead ratio was 100 ± 0.45%, 99.96 ± 0.19%, 99.85 ± 0.17%, 98.87 ± 0.30% and 98.62 ± 0.39% for normal group, and Ce@P + NIR for 0, 5, 10 and 15 min (Fig. S5). Thus, 5 min was applied for further experiments during NIR irradiation.

In addition, for ALI therapy, NPs ultimately need to enter the circulatory system. Thus, it was expected that NPs possessed good blood compatibility. The hemolysis test was implemented by incubating fresh blood with NPs of various concentrations. As displayed in Fig. S6, the evident hemolysis happened for DI water while no hemolysis was observed for Ce@P with the concentrations ranging from 5 to 500 µg/mL. By statistical analysis, the hemolysis ratio was below 5% for all groups during this concentration ranges, indicating that Ce@P possessed excellent hemocompatibility.

### Cellular uptake ability

In order to fully utilize the efficacy of NPs, it was anticipated that NPs could be uptake by the targeted cells and the corresponding biological functions occurred within cells. Due to the thiol group of Cy5-PEG2000-Thiol, it could react with metal bonds of CeO_2_, and metal bonds and double bonds of Ce@P to form stable Cy5 labeled NPs (Cy5-CeO_2_ and Cy5-Ce@P). Herein, Cy5-CeO_2_ and Cy5-Ce@P were incubated with cells for 3 h respectively, followed by the observation of confocal microscope. As shown in Fig. 4D and E, compared to Cy5-CeO_2_ with a little of fluorescence observed, the obvious fluorescence existed for Cy5-Ce@P. It confirmed that it was easy to be uptake by cells for Ce@P, exerting its intracellular biological functions. Compared to CeO_2_, cellular uptake capacity was enhanced for Ce@P, consistent with the previously reported results [48]. Although the zeta potential of Ce@P changed from positive to negative charge, PDA coating could improve the adhesive ability of Ce@P, helpful to strength cellular uptake capacity [37, 48].

### Intracellular ROS levels

**Fig. 5:**
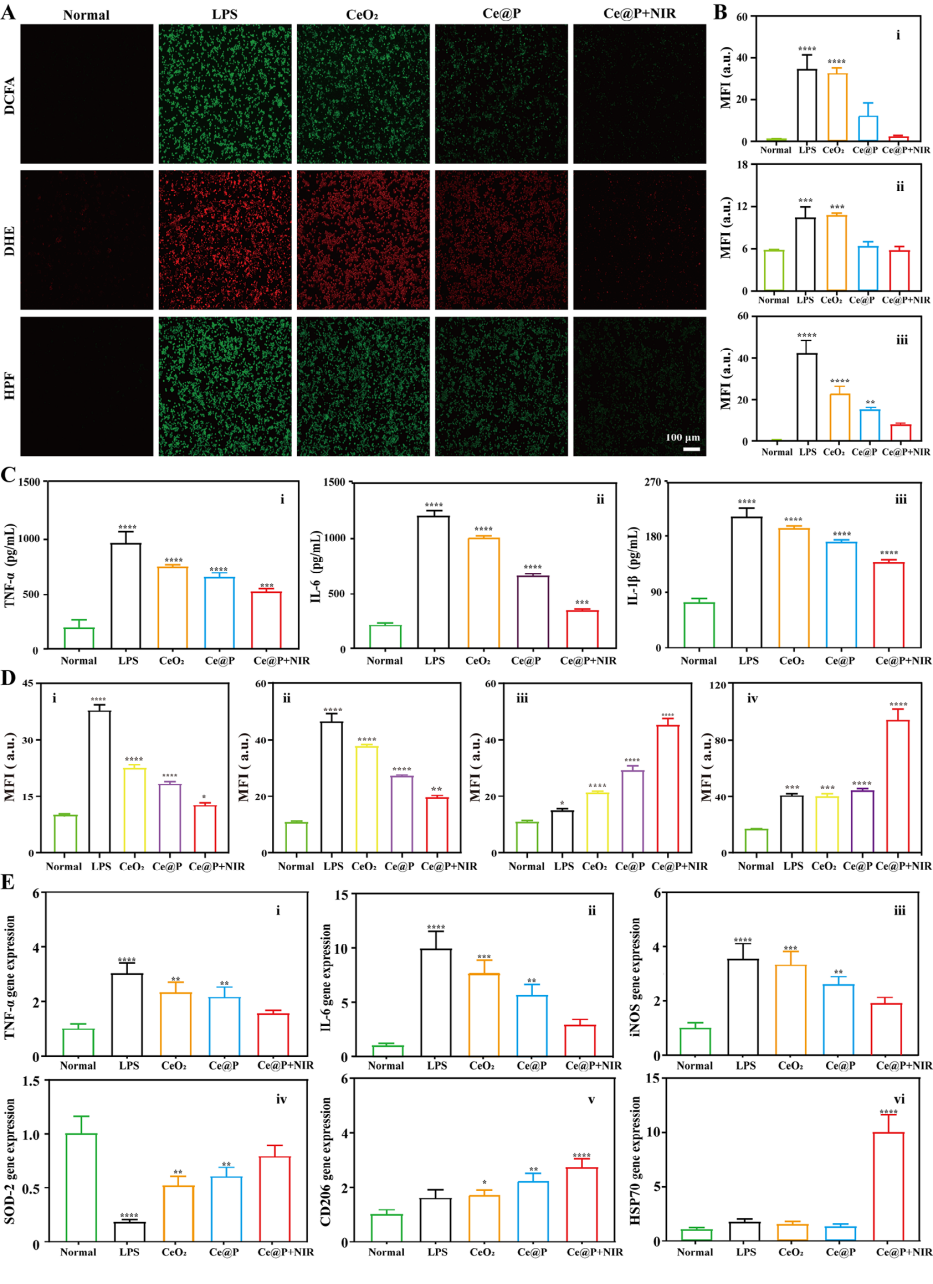
Antioxidant and anti-inflammation capacity in cellular levels. **A**) Intracellular ROS (DCFA, DHE and HPF) levels of treated cells by fluorescent microscope and the corresponding quantified results (DCFA (i), DHE (ii) and HPF (iii)) (**B**). **C**) Inflammatory factors (TNF-α (i), IL-6 (ii) and IL-1β (iii)) expression levels of the supernatent of treated cells by ELISA. **D**) Quantified results of IL-6 (i), iNOS (ii), CD206 (iii) and HSP70 (iv) expression level of treated cells by fluorescent microscope. F) Inflammatory and anti-inflammatory genes (TNF-α (i), IL-6 (ii), iNOS (iii), SOD-2 (iv) and CD206 (v)), and HSP70 (vi) gene expression levels of treated cells by qRT-PCR. The corresponding groups were: cells without treatment (normal group), cells pre-treated with LPS followed by incubating with PBS buffer (LPS group), cells pre-treated with LPS followed by incubating with 100 µg/mL CeO_2_ (CeO_2_), cells pre-treated with LPS followed by incubating with 100 µg/mL Ce@P (Ce@P), and cells pre-treated with LPS followed by incubating with 100 µg/mL Ce@P and NIR irradiation (1.5 W/cm^2^) (Ce@P + NIR). (“*” symbol compared with normal group, *p < 0.05, **p < 0.01, ***p < 0.001 and ****p < 0.0001)

The intracellular ROS levels were investigated by utilizing ROS probes including DCFA, DHE and HPF. As shown in Fig. 5A, after LPS induction, the total ROS levels (DCFA) were enhanced for LPS group compared with normal group, confirmed by the enhanced fluorescent intensity. However, CeO_2_ only slightly decreased the total ROS levels while it significantly descended for Ce@P. Significantly, almost no fluorescence was observed for Ce@P with NIR irradiation, close to normal cells, indicating high scavenging capacity of total ROS levels. By statistical analysis, the corresponding mean fluorescence intensity (MFI) of normal cells was 0.98 ± 0.08, which increased to 34.47 ± 6.72 for LPS group, 32.50 ± 2.49 for CeO_2_, 12.08 ± 6.20 for Ce@P, and 2.15 ± 0.06 for Ce@P + NIR respectively (i of Fig. 5B). Similarly, it presented the same tendency for intracellular ·O_2_^-^ (DHE, ii of Fig. 5B) and ·OH (HPF, iii of Fig. 5B) levels, with the order of control groupincreasing NIR irradiation intensityCeO_2_>Ce@P>Ce@P + NIR>normal group. Ce@P + NIR presented the optimum capacity of lowering intracellular ROS levels, due to its best ROS scavenging capacity, further indicating the excellent antioxidant ability.

### Antioxidant and anti-inflammatory capacity

The antioxidant and anti-inflammatory capacities of NPs were initially assessed by utilizing ELISA to test the supernatant of cells. As displayed in Fig. 5C, LPS stimulation significantly increased the expression levels of inflammatory factors: TNF-α (i), IL-6 (ii) and IL-1β (iii), compared to normal group. CeO_2_ hardly affected the expression levels of TNF-α, IL-6 and IL-1β while Ce@P obviously decreased their expression levels. Significantly, Ce@P + NIR presented the optimum effects of lowering inflammatory factors expression levels.

Besides, the antioxidant and anti-inflammatory abilities were further evaluated by immunofluorescent staining. LPS stimulation significantly increased the fluorescent intensity of cells, indicating high expression of inflammatory protein (IL-6) (Fig. S7) and M1 type protein (iNOS) (Fig. S8) for LPS group, compared to normal cells. However, LPS stimulation decreased the expression of M2 type protein (CD206) (Fig. S9) with the MFI of 15.10 ± 0.75 while it was 11.03 ± 0.50 for normal cells. Nevertheless, Ce@P could efficiently decrease the expression levels of IL-6 (18.47 ± 0.88) and iNOS (27.37 ± 0.45), and increase the expression level of CD206 (29.40 ± 1.72) while CeO_2_ almost had no impacts on their expression levels, with the MFI of 22.67 ± 1.05, 38.87 ± 0.68 and 21.46 ± 0.53 respectively, close to those of normal cells (i, ii and iii of Fig. 5D). Most importantly, Ce@P + NIR possessed the weakest MFI of IL-6 and iNOS expression levels, and strongest MFI of CD206 expression level, indicating that it could most effectively inhibit the expression levels of inflammatory factors and induce M2 polarization of macrophages.

Previous studies demonstrated that HSP70 could restore the normal function of mutated proteins. And its high expression was helpful to promote tissue repair [49]. It also indicated that HSP70 possessed an anti-inflammatory function in macrophages [50, 51]. Thus, the expression level of HSP70 was also studied by immunofluorescent staining. As imaged in Fig. S10, the obvious high expression level of HSP70 existed in Ce@P + NIR compared to other groups. After statistical calculation, the MFI of HSP70 was 94.45 ± 8.11 for Ce@P + NIR, which decreased to 45.27 ± 1.71 for Ce@P, 40.94 ± 2.80 for CeO_2_, 41.78 ± 1.56 for LPS group, and 18.30 ± 0.51 for normal group respectively (iv of Fig. 5D).

At last, the antioxidant and anti-inflammatory abilities were also evaluated by RT-qPCR to analyze the levels of inflammatory genes: TNF-α, antioxidant genes: SOD2, M1 type gene: IL-6 and iNOS, and M2 type gene: CD206 (Fig. 5E). Consistent with the aforementioned immunofluorescence results, LPS stimulation could apparently ascend the expression levels of TNF-α (3.03±0.38), IL-6 (9.94 ± 1.61), iNOS (3.55 ± 0.57) and CD206 (1.61 ± 0.29), and descend SOD2 (0.18 ± 0.03) expression level (Fig. 5E). Nevertheless, incubating with NPs could lower the inflammatory genes and M1 type gene expression levels, and raise the anti-inflammatory genes and M2 type gene expression levels. Among them, it displayed the most effective regulation effects for Ce@P + NIR, followed by Ce@P and CeO_2_. Meanwhile, HSP70 gene was also analyzed by RT-qPCR (vi of Fig. 5E). Keeping up with the protein expression levels, Ce@P + NIR possessed the highest gene expression level of HSP70 (10.01 ± 1.59), followed by LPS group (1.66 ± 0.30), CeO_2_ (1.47 ± 0.24), Ce@P (1.26 ± 0.22), and normal group (1.01 ± 0.16).

Altogether, it demonstrated that Ce@P presented more effective antioxidant and anti-inflammatory capacities for LPS induced macrophages compared to CeO_2_, due to the fact that PDA coating contributed to enhanced ROS scavenging ability, and photothermal effects. Most significantly, compared to Ce@P, Ce@P + NIR could most effectively improve the protection ability of LPS induced cells, lowering the intracellular ROS levels, decreasing the expression levels of inflammatory factors and genes, up-regulating the expression levels of anti-inflammatory factors and genes, inducing directional polarization of macrophages from M1 to M2, as well as leading to high expression level of HSP70, all of which were beneficial for promoting the tissue repair.

### In vivo animal experiments

#### In vivo bio-distribution

**Fig. 6:**
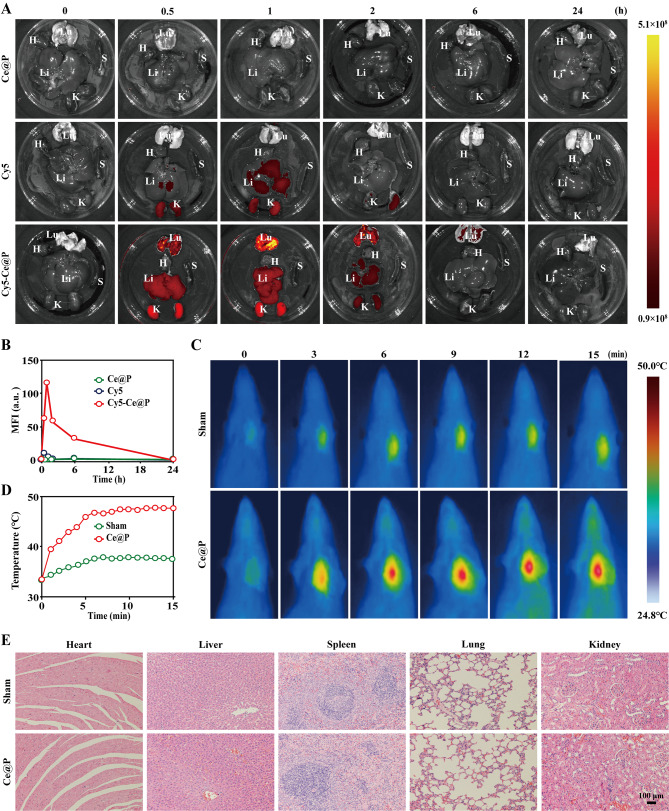
In vivo bio-distribution, photothermal effect and biosafety. **A**) Fluorescent images of major isolated organs (heart (H), liver (Li), spleen (S), lung (Lu) and kidney (K)) at predetermined time points (0, 0.5, 1, 2, 6 and 24 h) by IVIS and the corresponding quantified results of lung tissue (**B**). The corresponding groups were: rats injected with Ce@P (Ce@P), rats injected with Cy5 (Cy5), and rats injected with Cy5-Ce@P (Cy5-Ce@P). **C**) In vivo photothermal images of treated rats versus time under NIR irradiation (1.5 W/cm^2^) and the corresponding quantified results (**D**). The corresponding groups were: rat with PBS injection (sham group) and rat with Ce@P injection (Ce@P). **E**) H&E staining results of major organs of treated rats. The corresponding groups were: rats without treatment (sham group) and rats with Ce@P injection (Ce@P)

As biomedicines, it was anticipated that they could retain at the target location for a period of time, and ultimately undergo biodegradation and be cleared by human body. From the above in vitro results, it demonstrated that Ce@P possessed enhanced ROS scavenging ability and cellular uptake capacity together with photothermal conversion effect compared with CeO_2_. Thus, CeO_2_ was not considered to be applied for in vivo animal experiments. The in vivo bio-distribution of Ce@P was monitored by IVIS. As shown in Fig. 6A, after IV injection, almost no fluorescence occurred in isolated organs for Ce@P at predetermined time points. It demonstrated that Ce@P itself could not contribute fluorescent intensity. Besides, for Cy5 alone, it was possible to observe the fluorescent intensity in the liver and kidney while the fluorescence of liver and kidney disappeared at 2 h and 6 h respectively. However, there was no fluorescence existed in the lung at all monitoring time points. Specifically, the fluorescence was observed in lung, liver and kidney for Cy5-Ce@P. At 1 h, the fluorescent intensity reached maximum, indicating that Ce@P was most abundant in these organs during this time period. And the fluorescence still existed in the lung at 6 h, and disappeared after 24 h. From the statistical results, it was obviously shown that Ce@P gradually degraded in the body with the gradual decreased fluorescence versus time (Fig. 6B). From the above, it confirmed that Ce@P could stay in the lung for a certain of time, and eventually be cleared from the body. The differences observed between Cy5 and Cy5-Ce@P also gave the proof that the decreasing fluorescence of Cy5-Ce@P was attributed from the degradation of Ce@P rather than the quenching of Cy5 itself. Thus, it presented appropriate biodegradability for Ce@P, representing its great potentials in clinics.

### In vivo photothermal effect

It had confirmed that NIR irradiation could achieve spatiotemporal and controllable irradiation of disease areas, achieving the minimizing side effects [52–54]. Generally, the penetrate depth of tissue was around 1 ~ 2 cm for 808 nm NIR light [55]. To confirm the possibility of NIR irradiation for in vivo ALI therapy, in vivo photothermal effect was assessed by monitoring the temperature changes of lung site under NIR irradiation. After 1 h’ IV injection, the temperature was monitored by photothermal camera when NPs were enriched in the lung. As indicated in Fig. 6C, with the passage of irradiation, temperature changed from 33.80℃ to 37.80℃ for sham group. Nevertheless, the obvious temperature changes were observed for Ce@P versus time, jumping from 33.40℃ to 48.10℃ (Fig. 6D). Thus, Ce@P possessed significant in vivo photothermal effect, providing the feasibility of NIR driven enhanced in vivo ALI therapy.

### In vivo biosafety

To further confirm the feasibility of Ce@P for clinic application, in vivo biosafety was evaluated by investigating the body weight of rats, and blood indicators and pathological features of major organs after 7 day’ IV injection. As illustrated in Fig. S11, the body weight of normal rats (sham group) kept up with that of Ce@P, maintaining the same tendency within these 7 days. Similarly, as shown in Fig. S12 and Table S6, compared to sham group, there were no statistically significant in blood routine indicators (WBC, RBC, HGB and PLT), liver function indicators (AST and ALT), kidney function indicators (CREA and UREA), myocardial enzyme indicators (CK and CK-MB), and coagulation indicators (PT, INR, TT and FIB), confirmed by almost the similar numerical value observed. Meanwhile, from tissue sections, there were no damages for heart, liver, spleen, lung and kidney for Ce@P injection, close to sham group (Fig. 6E). From the above, it demonstrated that Ce@P presented outstanding in vivo biosafety, not affecting the body weight, blood indicators and pathological features. Thus, Ce@P showed promising prospects in clinic application.

### In vivo ALI therapy

As was known to all, sepsis was a multi-organs failure disease. To avoid damaging other organs, we chose intratracheal instillation of LPS to establish ALI animal model for further evaluations [45, 46]. And IV injection was applied to achieve in vivo ALI therapy due to the fact that the results of in vivo bio-distribution and blood biocompatibility of NPs supported the feasibility of high efficacy ALI therapy. Thus, after establishing ALI models, the rats were IV injected with the corresponding solutions. And blood samples and major organs including heart, liver, spleen, lung and kidney were collected for further experiments after 24 h. In vivo therapy was initially evaluated by investigating the change of blood indicators including blood routine indicators, liver function indicators, kidney function indicators, myocardial enzyme indicators, and coagulation indicators. From Fig. S13 and Table S7, no significant differences of relative indicators were observed in all groups except for AST and ALT, which significantly increased in ALI group, and decreased in Ce@P and Ce@P + NIR.

**Fig. 7:**
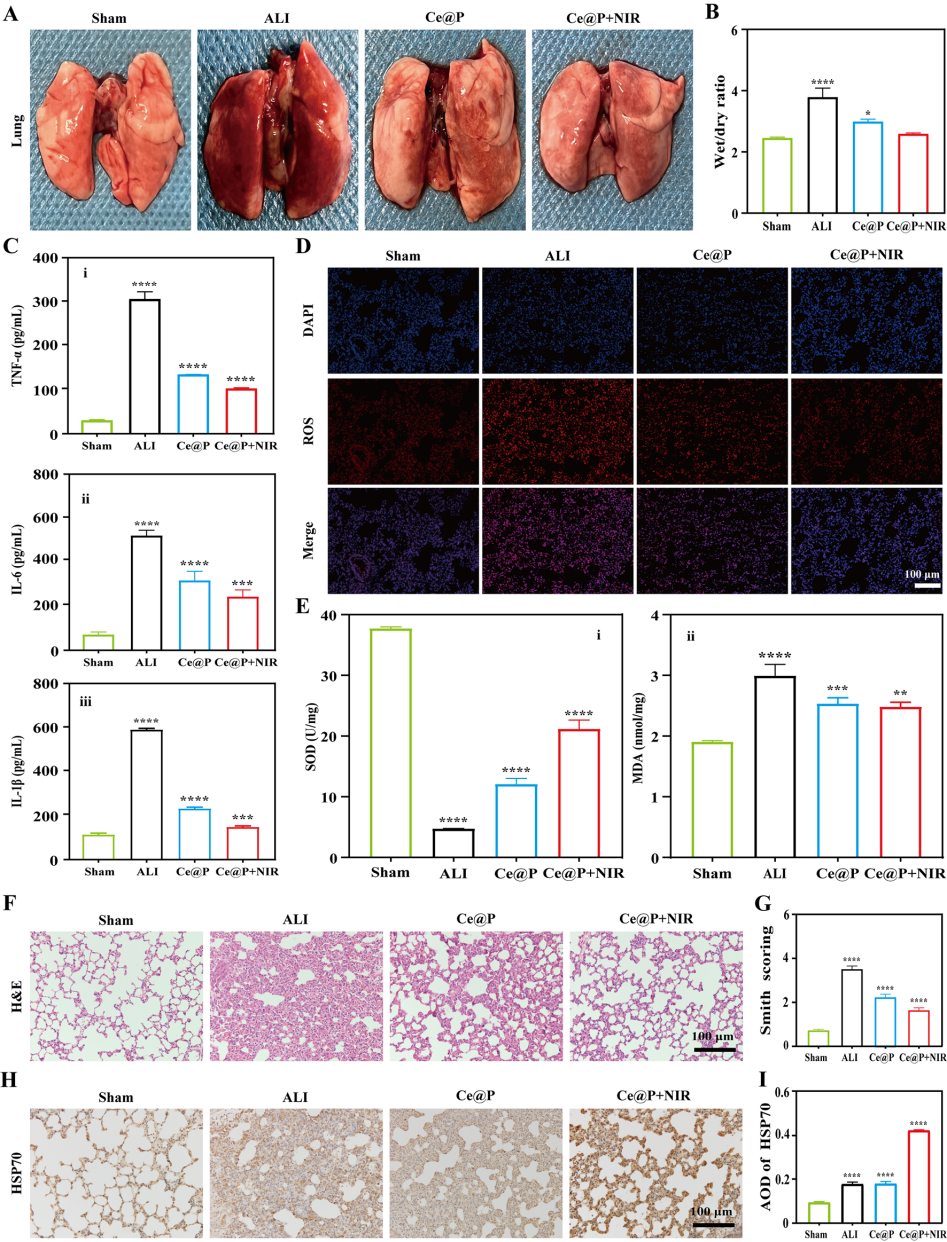
In vivo ALI therapy evaluation. **A**) Macroscopic observation of lung tissue of treated rats. **B**) The wet/dry ratio of lung tissue of treated rats. **C**) Inflammatory factors (TNF-α (i), IL-6 (ii) and IL-1β (iii)) expression levels of lung homogenate of treated rats by ELISA. **D**) ROS staining images of lung tissue of treated rats. **E**) SOD (i) and MDA (ii) levels of lung homogenate of treated rats by the corresponding testing kits. **F**) H&E staining images of lung tissue of treated rats and the corresponding Smith score (**G**). **H**) HSP70 expression level of lung tissue of treated rats by immunohistochemical staining and the corresponding average optical density (AOD) (I). The corresponding groups were: rats without treatment (sham group), LPS induced rats with PBS injection (ALI group), LPS induced rats with Ce@P injection (Ce@P) and LPS induced rats with Ce@P injection and NIR irradiation (Ce@P + NIR). (“*” symbol compared with normal group, *p < 0.05, **p < 0.01, ***p < 0.001 and ****p < 0.0001)

The gross view of lung tissues was observed. As revealed in Fig. 7A, both lungs were in pink color with smooth surface and good elasticity for sham group. However, both lungs were congested in dark red color, with partial visible patchy necrosis. After Ce@P injection, the congestion of both lungs was alleviated, significantly enhanced for Ce@P + NIR. At the same time, the wet/dry (w/d) ratios of lungs were also evaluated. As listed in Fig. 7B, the w/d ratio of the lung was 2.45 ± 0.03 for sham group, increased to 3.78 ± 0.30 for ALI group. Nevertheless, it decreased to 2.99 ± 0.08 and 2.59 ± 0.03 for Ce@P and Ce@P + NIR respectively.

Next, the inflammatory factors levels of lung tissues were analyzed by ELISA. For ALI group, the levels of IL-6, TNF-α and IL-1b significantly ascended to 514.00 ± 24.55, 311.60 ± 17.32 and 609.60 ± 6.76 pg/mL respectively compared to sham group only with 66.39 ± 12.26, 30.08 ± 1.65 and 116.70 ± 7.50 pg/mL (Fig. 7C). Ce@P injection could effectively decrease IL-6, TNF-α and IL-1b expression levels to 316.20 ± 41.88, 135.50 ± 0.34 and 236.40 ± 6.71 pg/mL. Most importantly, with NIR irradiation, the corresponding IL-6, TNF-α and IL-1b expression levels were obviously declined for Ce@P + NIR, with 243.00 ± 31.31, 102.20 ± 1.94 and 151.30 ± 5.59 pg/mL separately.

In addition, the ROS levels of lung tissue were also tested by immunofluorescent staining. Compared to sham group, the significant fluorescence was observed for ALI group, indicating high ROS levels after LPS induction. Ce@P could lower ROS levels with decreased fluorescence observed, significantly enhanced by Ce@P with NIR irradiation (Fig. 7D). Meanwhile, MDA and SOD levels were also evaluated for all groups. As indicated in Fig. 7E, compared to normal group, SOD level decreased to 4.60 ± 0.05 U/mg while MDA level ascended to 2.98 ± 0.19 nmol/mg for ALI group. However, the corresponding SOD level and MDA level were 11.95 ± 0.93 U/mg and 2.53 ± 0.10 nmol/mg for Ce@P, and 20.91 ± 1.56 U/mg and 2.47 ± 0.08 nmol/mg for Ce@P + NIR respectively.

Furthermore, the pathology analysis of lung tissue was evaluated. As presented in Fig. 7F, there was no significant infiltration of inflammatory cells in alveoli and alveolar interstitium with normal structure of lung tissue for sham group. However, for ALI group, the lung structure was disordered with visible thicken alveolar wall, and a lot of neutrophil infiltration was found. And it was obviously observed that the alveolar wall became thin with few visible inflammatory cells for Ce@P + NIR, better than those of Ce@P. After Smith scoring, it was 0.73 ± 0.06, 3.57 ± 0.15, 2.27 ± 0.15 and 1.67 ± 0.12 for sham group, ALI group, Ce@P and Ce@P + NIR respectively (Fig. 7G). Most importantly, the expression level of HSP70 was also evaluated. Compared to other groups including Ce@P, it displayed the highest expression level of HSP70 for Ce@P + NIR, helpful to promote the repair of lung tissue (Fig. 7H and I).

Ultimately, the other organs were also evaluated by gross view and H&E staining. It displayed no damages in all other organs (heart, liver, spleen and kidney) for ALI group, Ce@P and Ce@P + NIR respectively, close to sham group (Fig. S13 and S14).

In conclusion, it had confirmed that Ce@P + NIR presented the optimum in vivo ALI therapy effect. Previous study had confirmed that PDA served as a nanozyme with excellent ROS scavenging capacity, and also acted as a photothermal agent, achieving efficient photothermal conversion [32, 36]. Herein, the enhanced ROS scavenging capacity was achieved by combining CeO_2_ with PDA, also equaled to lowering the inflammatory factors expression levels of lung tissue, decreasing ROS and MDA levels and increasing SOD level of the lung, and alleviating lung tissue damage. Significantly, with NIR irradiation, it not only contributed to enhanced ROS scavenging capacity, but also had promoted the expression level of HSP70, helpful to accelerate the repair of lung tissue. All the previous results gave the proof that Ce@P combining with NIR irradiation could most effectively achieve ALI therapy by excellent ROS scavenging and photothermal effect.

## Conclusion

In this study, we successfully developed a novel nanozyme (Ce@P) for the synergistic enhanced ALI therapy. The nanozyme was designed by encapsulating CeO_2_ with PDA coating, thus realizing the combination of ROS scavenging and photothermal therapy. PDA coating not only enabled Ce@P to serve as a photothermal agent, but also offered the enhanced ROS scavenging capacity. The excellent ROS scavenging, combined with photothermal enhancement, achieved an efficient synergistic ALI therapy by down-regulating the expression level of inflammatory cytokines, decreasing the intracellular ROS levels, and inducing M2 directional polarization. Notably, Ce@P could significantly be accumulated in the lung by IV injection, and gradually degraded versus time, displaying excellent biocompatibility and hemocompatibility. Combining with NIR stimulation, it presented the outstanding behaviors of decreasing lung inflammation, alleviating diffuse alveolar damage, and promoting the expression level of HSP70 in the lung. This work concluded that Ce@P + NIR, a novel therapy strategy with synergistic enhancement of ALI therapy, show promising potentials in clinical treatment of ROS derived diseases.

### Electronic supplementary material

Below is the link to the electronic supplementary material.

Supplementary Material 1

Supplementary Material 2

## Data Availability

No datasets were generated or analysed during the current study.
