# Supplementary material for: NIR triggered polydopamine coated cerium dioxide nanozyme for ameliorating acute lung injury via enhanced ROS scavenging
Source: J Nanobiotechnology. 2024 Jun 8;22:321. doi: 10.1186/s12951-024-02570-w (PMC11162040; doi:10.1186/s12951-024-02570-w)
Supplement: Supplementary file 1 — Supplementary Material 1 [file 12951_2024_2570_MOESM1_ESM.docx]

Supplementary Materials

**NIR triggered polydopamine coated cerium dioxide nanozyme for ameliorating acute lung injury via enhanced ROS scavenging**

Mingjing Yin ^a #^, Doudou Lei ^a, b #^, Yalan Liu ^a #^, Tao Qin ^c^, Huyang Gao ^b^, Wenquan Lv ^d^, Qianyue Liu ^b^, Lian Qin ^a^, Weiqian Jin ^b^, Yin Chen ^a^, Hao Liang ^e^, Bailei Wang ^a^, Ming Gao ^b *^, Jianfeng Zhang ^a *^ and Junyu Lu ^a *^

^a^ Intensive Care Unit, The Second Affiliated Hospital of Guangxi Medical University, Nanning, Guangxi 530007, China

^b^ Life Sciences Institute, Guangxi Medical University, Nanning, Guangxi 530021, China

^c^ Department of Intensive Care Unit, Guangxi Medical University Cancer Hospital, Nanning, Guangxi 530021, China

^d^ Department of Emergency, Guangxi Hospital Division of The First Affiliated Hospital, Sun Yat-sen University, Nanning, Guangxi 530022, China

^e^ College & Hospital of Stomatology, Guangxi Medical University, Nanning, Guangxi 530021, China


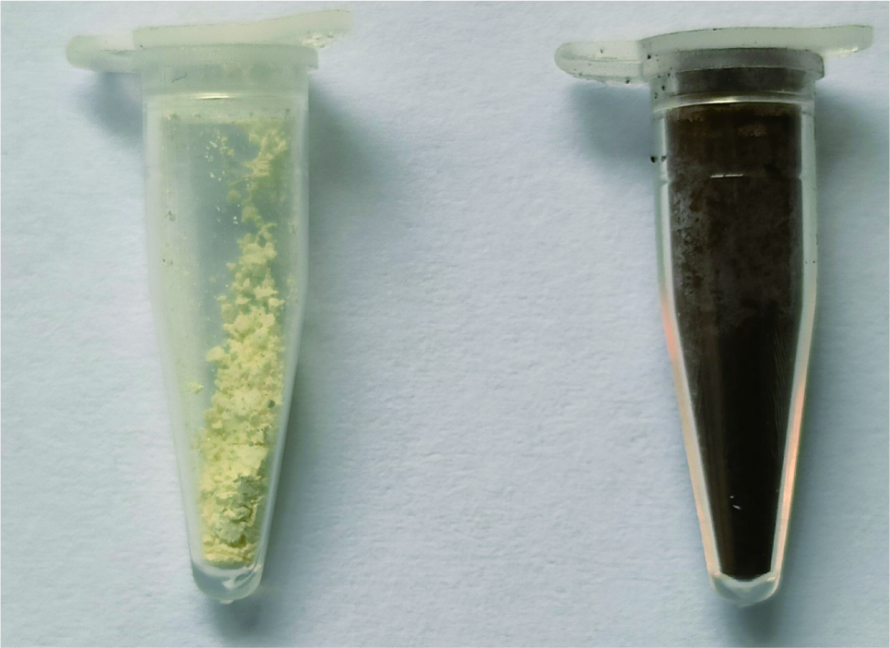


Fig. S1. Optical image of CeO_2_ and Ce@P (from left to right).


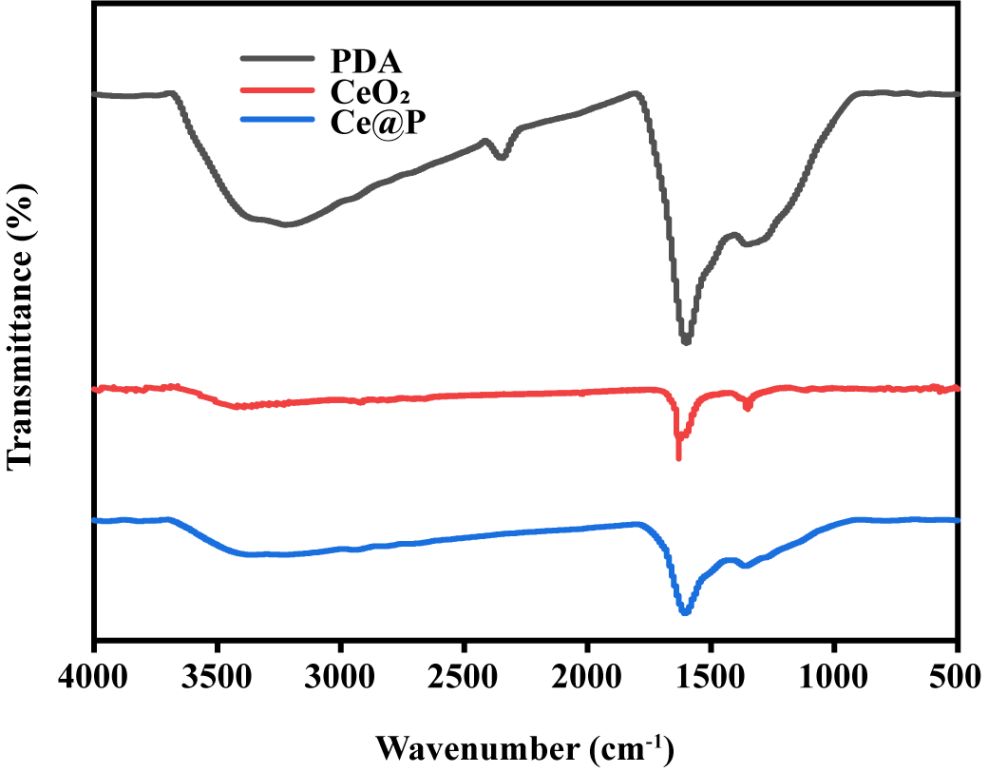


Fig. S2. FTIR results of CeO_2_ and Ce@P.


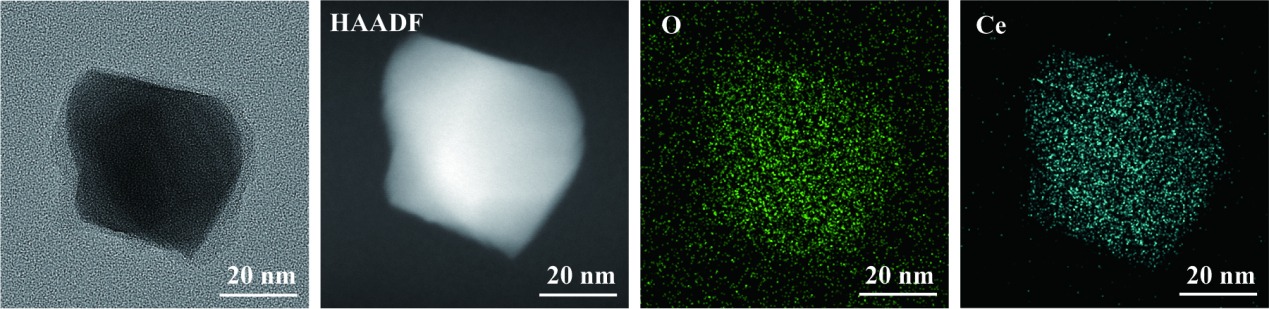


Fig. S3. TEM-mapping results of CeO_2_ and the corresponding element composition (HAADF, Ce and O images).


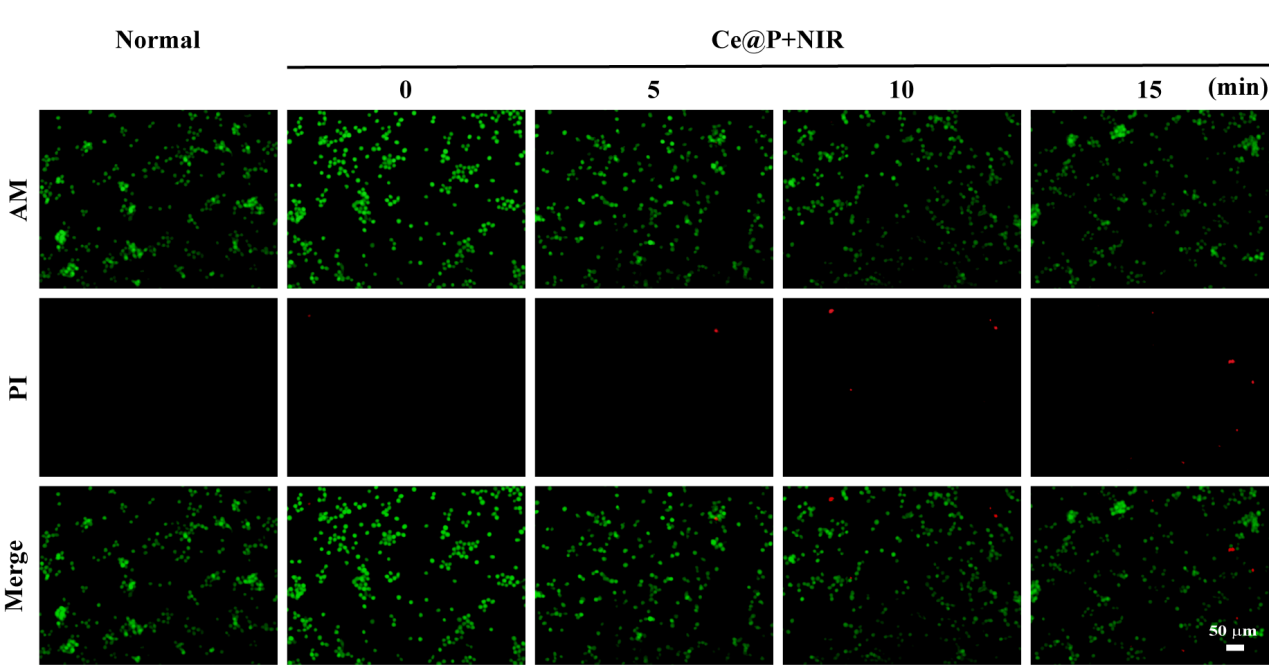
Fig. S4. Live/dead staining images of cells after Ce@P incubation under NIR irradiation for predetermined time points. The corresponding groups were: cells without treatment (normal group), cells incubating with Ce@P under NIR irradiation for 0, 5, 10 and 15 min.


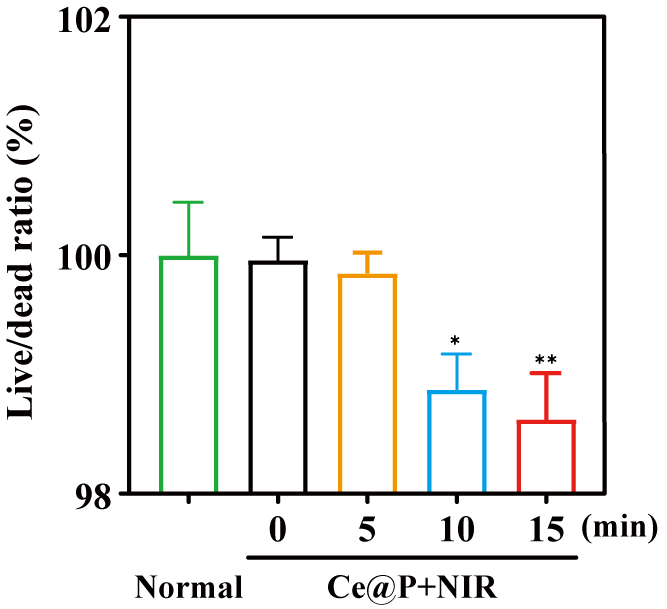


Fig. S5. The corresponding quantified results of Fig. 4. (“*” symbol compared with normal group, *p<0.05 and **p<0.01)


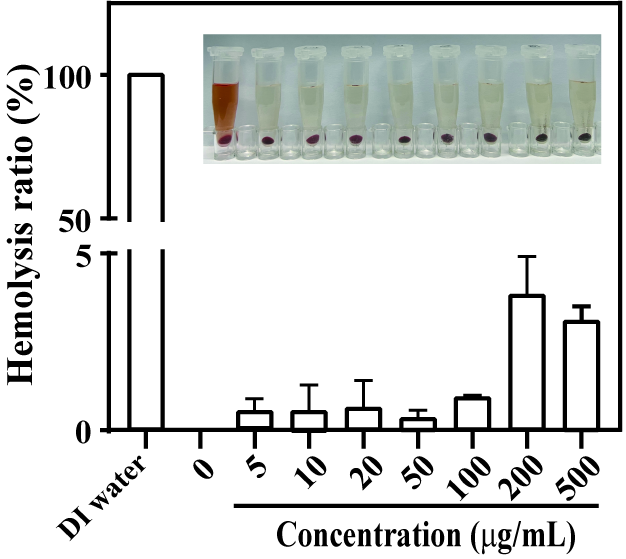


Fig. S6. Hemolysis ratio of Ce@P with different concentrations ranging from 0 to 500 μg/mL (The inserted images were DI water, 0, 5, 10, 20, 50, 100, 200 and 500 μg/mL Ce@P (from left to right)).


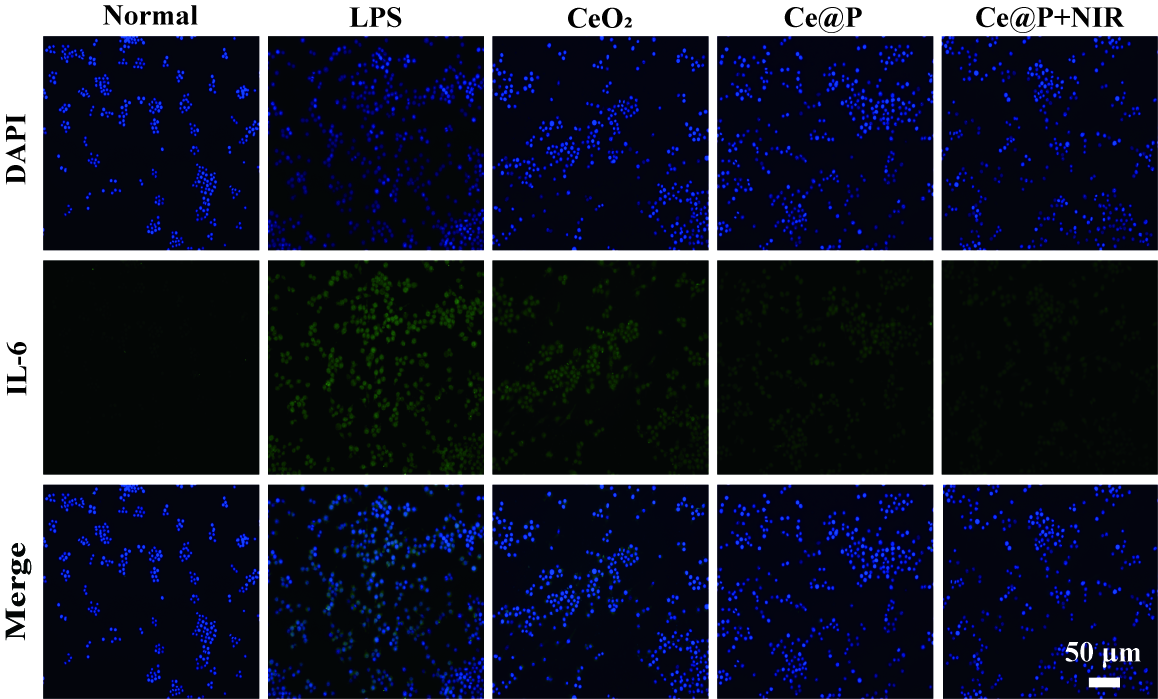


Fig. S7. IL-6 expression level of treated cells by fluorescent microscope. The corresponding groups were: cells without treatment (normal group), cells pre-treated with LPS followed by incubating with PBS buffer (LPS group), cells pre-treated with LPS followed by incubating with 100 μg/mL CeO_2_ (CeO_2_), cells pre-treated with LPS followed by incubating with 100 μg/mL Ce@P (Ce@P), and cells pre-treated with LPS followed by incubating with 100 μg/mL Ce@P and NIR irradiation (1.5 W/cm^2^) (Ce@P+NIR).


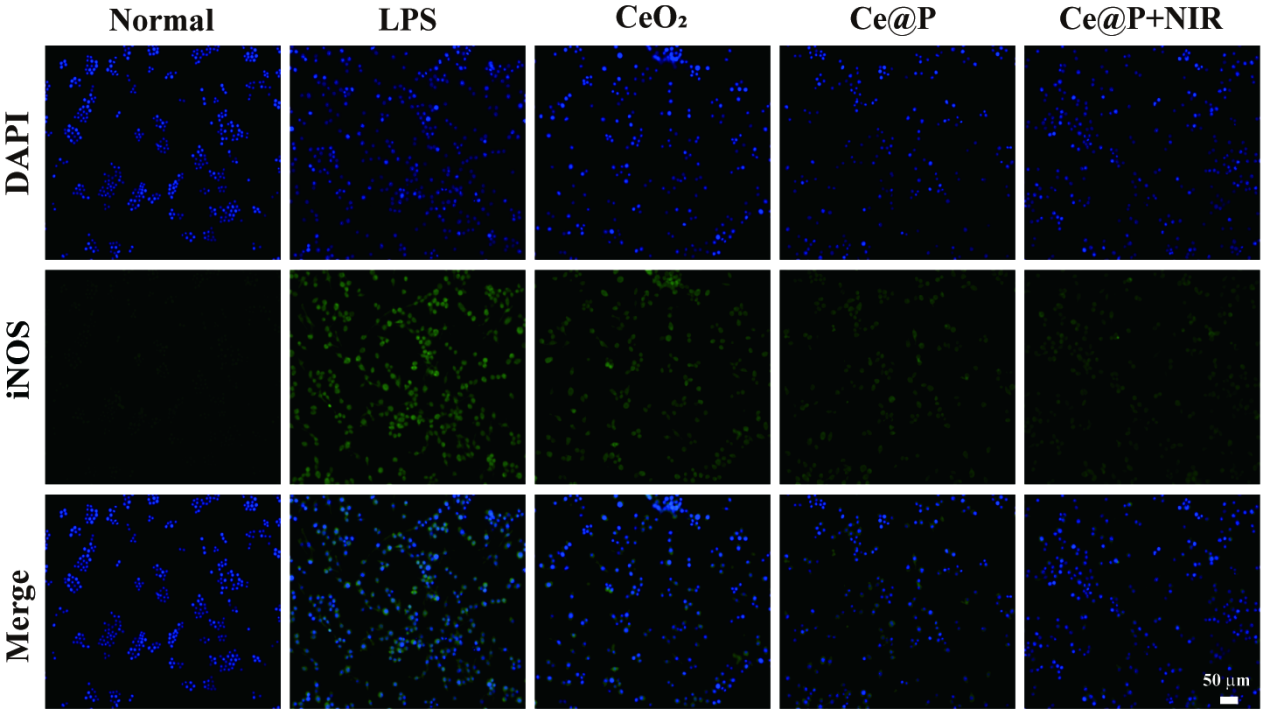


Fig. S8. iNOS expression level of treated cells by fluorescent microscope. The corresponding groups were: cells without treatment (normal group), cells pre-treated with LPS followed by incubating with PBS buffer (LPS group), cells pre-treated with LPS followed by incubating with 100 μg/mL CeO_2_ (CeO_2_), cells pre-treated with LPS followed by incubating with 100 μg/mL Ce@P (Ce@P), and cells pre-treated with LPS followed by incubating with 100 μg/mL Ce@P and NIR irradiation (1.5 W/cm^2^) (Ce@P+NIR).


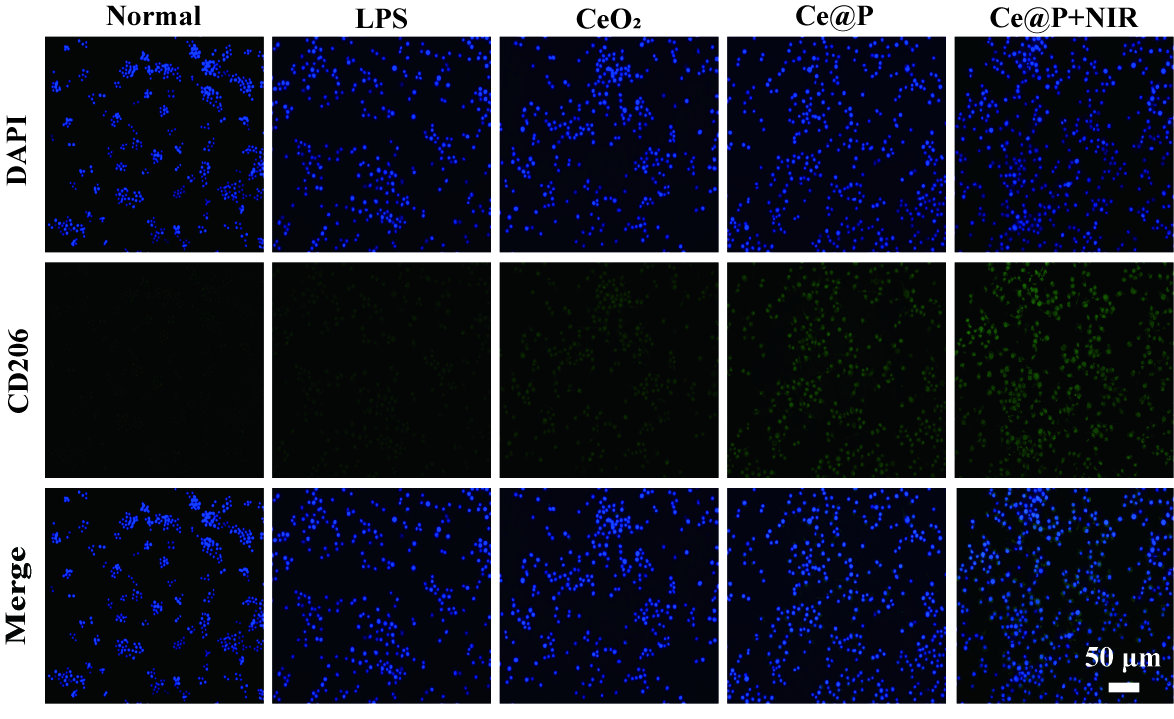


Fig. S9. CD206 expression level of treated cells by fluorescent microscope. The corresponding groups were: cells without treatment (normal group), cells pre-treated with LPS followed by incubating with PBS buffer (LPS group), cells pre-treated with LPS followed by incubating with 100 μg/mL CeO_2_ (CeO_2_), cells pre-treated with LPS followed by incubating with 100 μg/mL Ce@P (Ce@P), and cells pre-treated with LPS followed by incubating with 100 μg/mL Ce@P and NIR irradiation (1.5 W/cm^2^) (Ce@P+NIR).


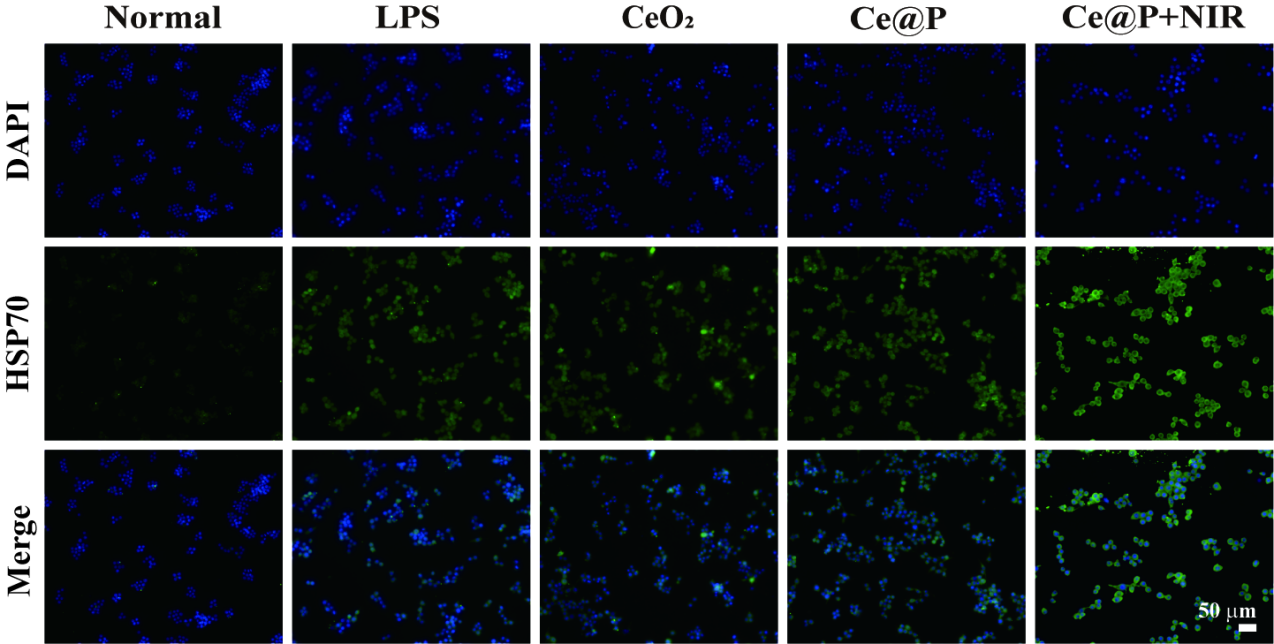


Fig. S10. HSP70 expression level of treated cells by fluorescent microscope. The corresponding groups were: cells without treatment (normal group), cells pre-treated with LPS followed by incubating with PBS buffer (LPS group), cells pre-treated with LPS followed by incubating with 100 μg/mL CeO_2_ (CeO_2_), cells pre-treated with LPS followed by incubating with 100 μg/mL Ce@P (Ce@P), and cells pre-treated with LPS followed by incubating with 100 μg/mL Ce@P and NIR irradiation (1.5 W/cm^2^) (Ce@P+NIR).


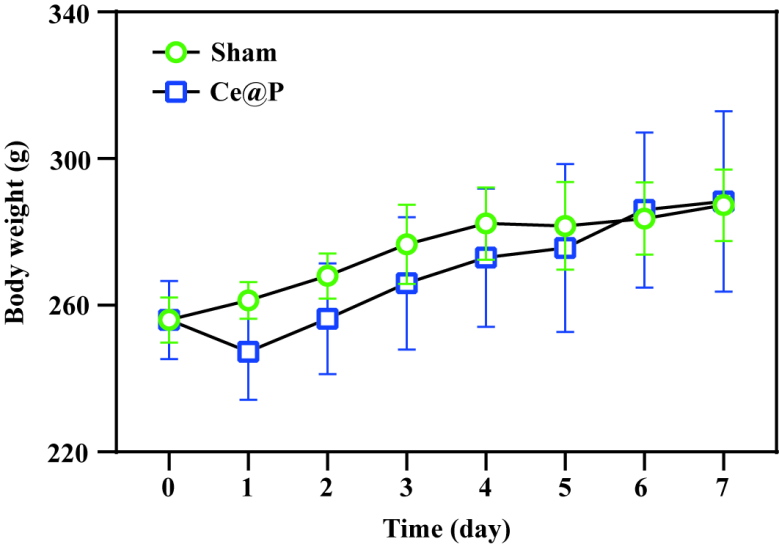


Fig. S11. The body weight change of treated rats for 7 day. The corresponding groups were: rats with PBS injection (sham group) and rats with Ce@P injection (Ce@P).


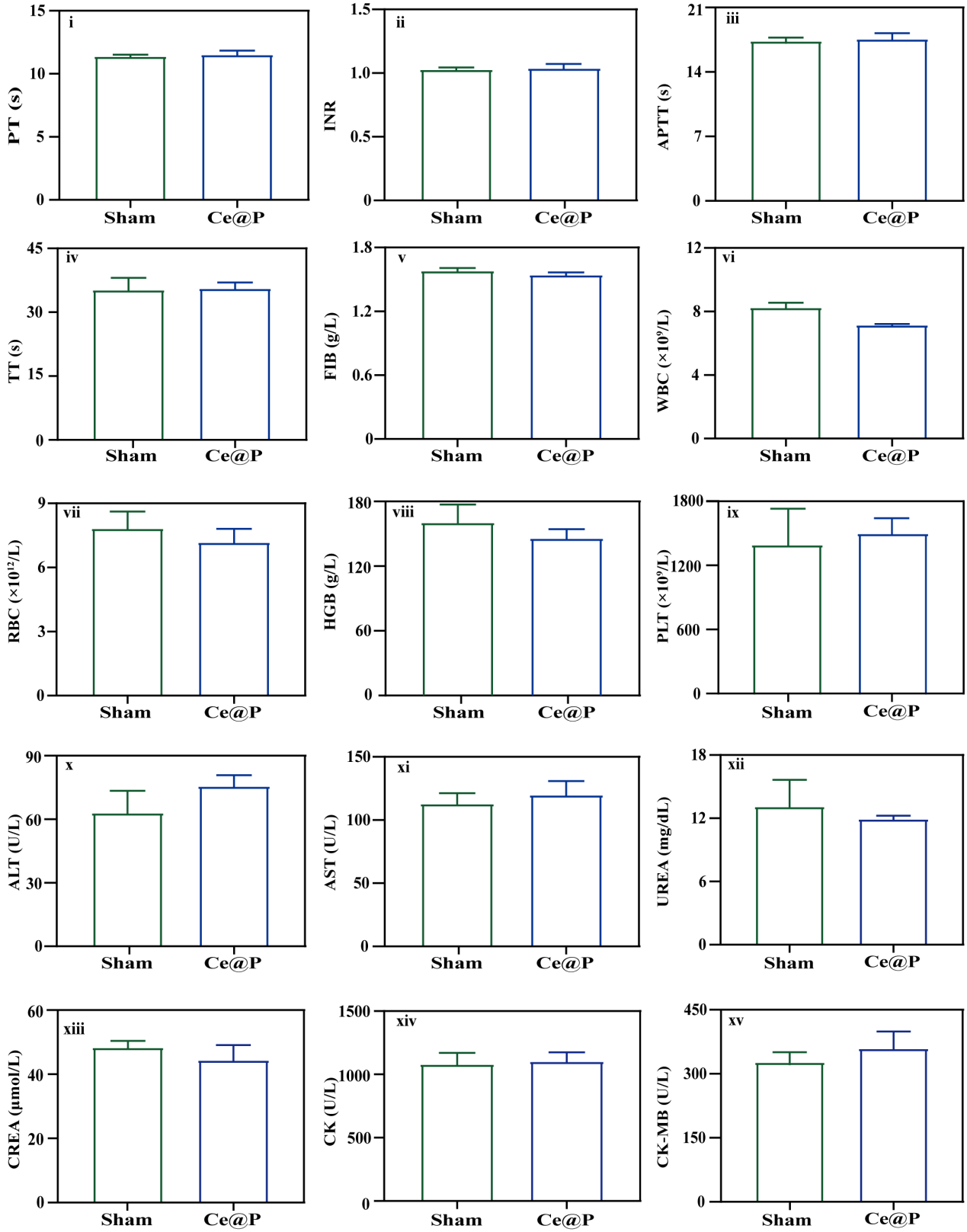


Fig. S12. Blood indicators of treated rats after 7 day. The corresponding groups were: rats with PBS injection (sham group) and rats with Ce@P injection (Ce@P).


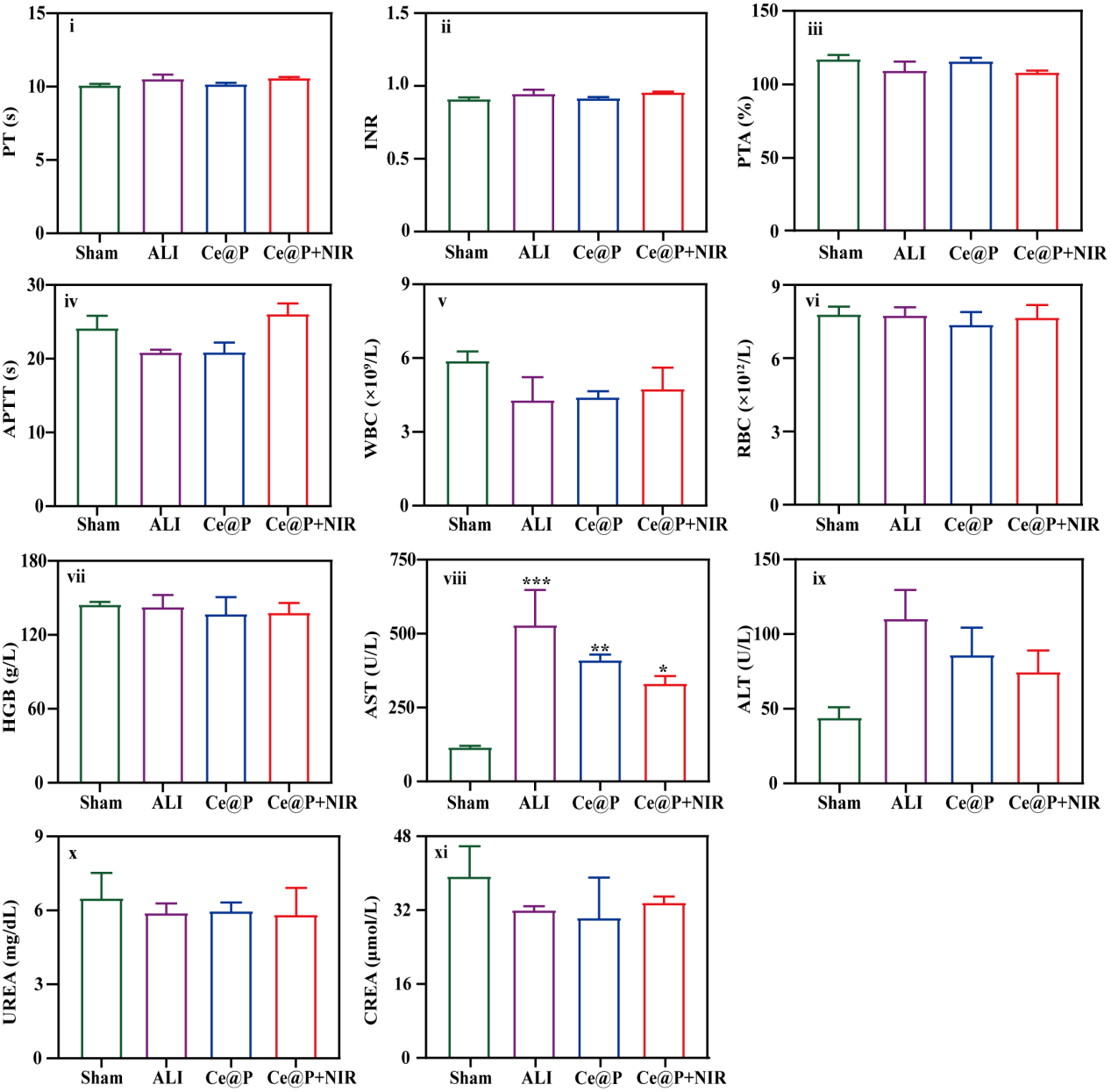


Fig. S13. Blood indicators of treated rats. The corresponding groups were: rats without treatment (sham group), LPS induced rats with PBS injection (ALI group), LPS induced rats with Ce@P injection (Ce@P) and LPS induced rats with Ce@P injection combining with NIR irradiation (Ce@P+NIR). (“*” symbol compared with sham group, *p<0.05, **p<0.01 and ***p<0.001)


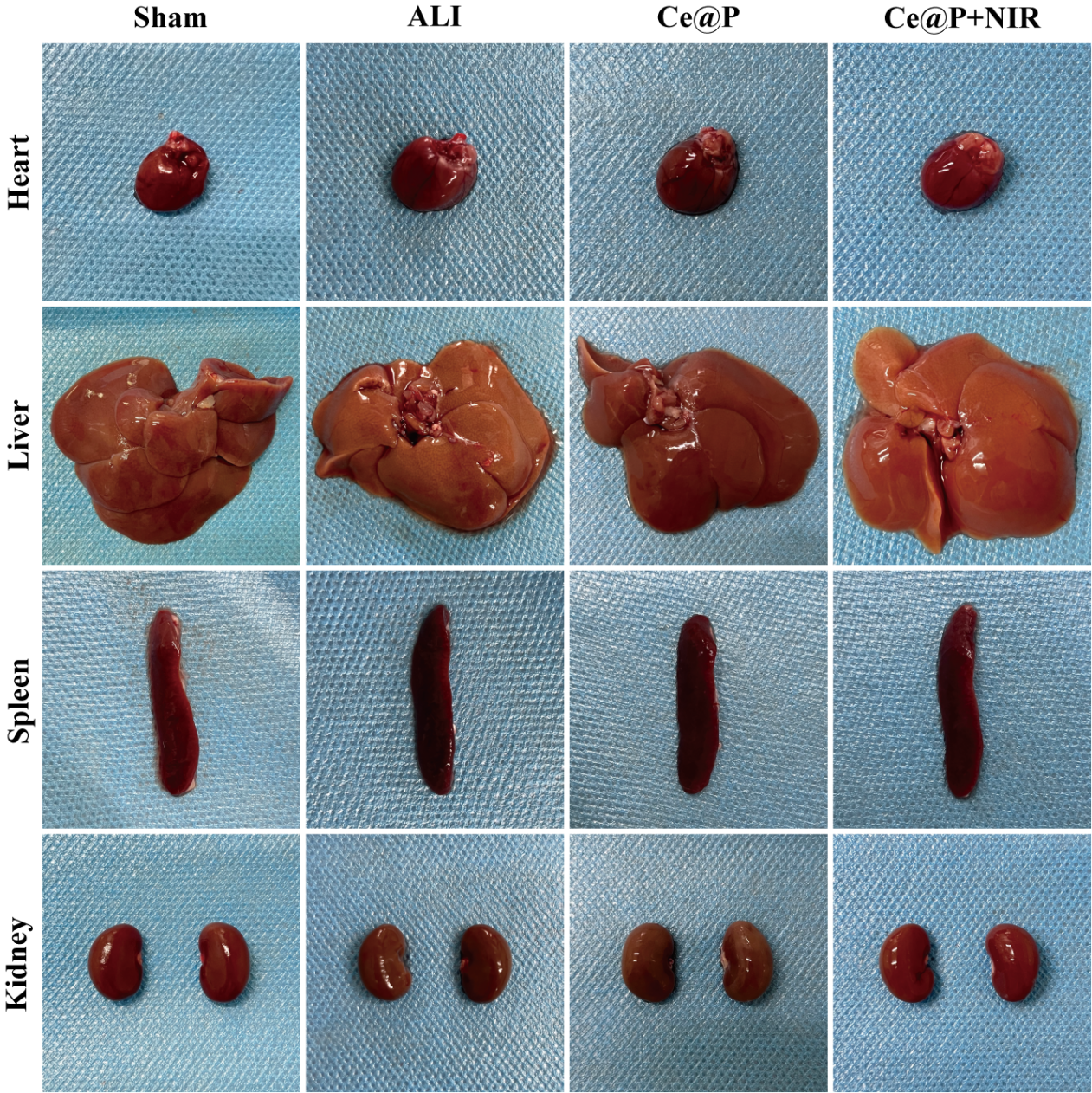


Fig. S14. Macroscopic observation of other tissues (heart, liver, spleen and kidney) of treated rats. The corresponding groups were: rats without treatment (sham group), LPS induced rats with PBS injection (ALI group), LPS induced rats with Ce@P injection (Ce@P) and LPS induced rats with Ce@P injection combining with NIR irradiation (Ce@P+NIR).


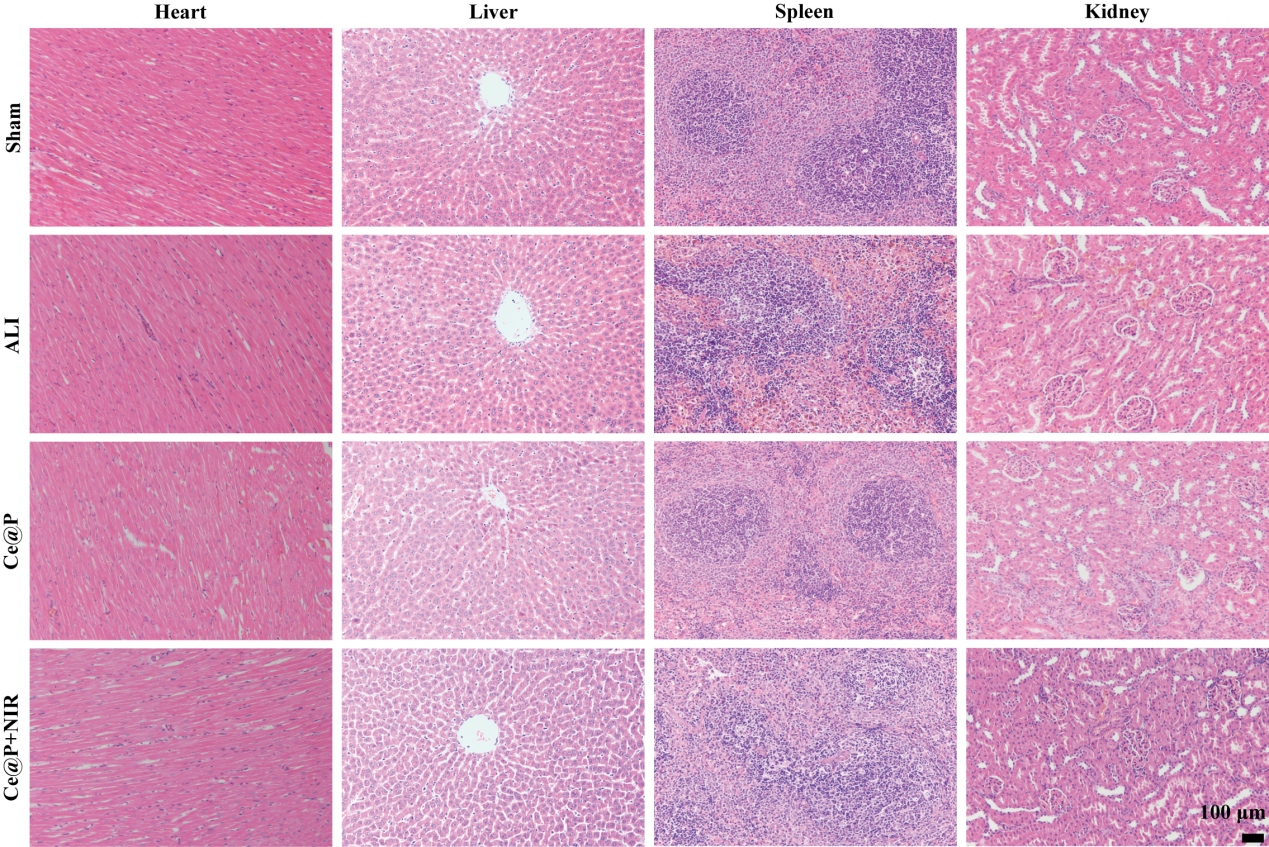


Fig. S15. H&E staining images of other tissues (heart, liver, spleen and kidney) of treated rats. The corresponding groups were: rats without treatment (sham group), LPS induced rats with PBS injection (ALI group), LPS induced rats with Ce@P injection (Ce@P) and LPS induced rats with Ce@P injection combining with NIR irradiation (Ce@P+NIR).
