# Supplementary material for: NIR triggered polydopamine coated cerium dioxide nanozyme for ameliorating acute lung injury via enhanced ROS scavenging
Source: J Nanobiotechnology. 2024 Jun 8;22:321. doi: 10.1186/s12951-024-02570-w (PMC11162040; doi:10.1186/s12951-024-02570-w)
Supplement: Supplementary file 2 — Supplementary Material 2 [file 12951_2024_2570_MOESM2_ESM.docx]

Table S1. Reaction recipe of Ce@P and the corresponding zeta potential.

| Name | CeO_2_ (g) | DA (g) | N_2_H_4_·H_2_O (mL) | Zeta potential (mV) |
| --- | --- | --- | --- | --- |
| CeO_2_ | / | / | / | 33.17 ± 1.38 |
| Ce@P | 1.00 | 0.20 | 0.10 | -28.50 ± 0.79 |

Table S2. Primer sequences for qRT-PCR.

| Gene | Forward sequences (5’ to 3’) | Reverse sequences (3’ to 5’) |
| --- | --- | --- |
| ACTB | CAGCCTTCCTTCTTGGGTAT | GGTCTTTACGGATGTCAACG |
| IL-6 | AGCTGGAGTCACAGAAGGAG | CTTAGGCATAACGCACTAGG |
| TNF-α | GAAGGGAATGGGTGTTCATC | TCACTGTCCCAGCATCTTGT |
| SOD2 | GTAGGGCCTGTCCGATGATG | CGCTACTGAGAAAGGTGCCA |
| iNOS | TGGAGCGAGTTGTGGATTGT | GTGAGGGCTTGGCTGAGTGA |
| CD206 | GTGATGGTTCTCCCGTTTCC | TGTGGGCTCAGGTAGTAGTG |
| HSP70 | CTTGGGCACCGATTACTGTC | ATAATCCCCTGGTACAGTGC |

Table S3. Element composition of CeO_2_ and Ce@P by XPS and ICP-MS.

| Name | XPS (%) | | | | ICP-MS (%) |
| --- | --- | --- | --- | --- | --- |
|  | C | N | O | Ce | Ce |
| CeO_2_ | 36.14 | 0 | 48.91 | 14.95 | / |
| Ce@P | 58.67 | 6.65 | 30.44 | 4.23 | 32.20 ± 0.29 |

Table S4. ROS scavenging ratio of different nanozymes with the same concentration of 100 μg/mL.

| Name | CeO_2_ (%) | Ce@P (%) | Ce@P + NIR (%) |
| --- | --- | --- | --- |
| H_2_O_2_ | 14.27 ± 1.60 | 44.63 ± 0.33 | 76.70 ± 0.12 |
| ·OH | 32.20 ± 2.02 | 40.58 ± 0.51 | 63.50 ± 1.92 |
| ·O_2_^−^ | 46.21 ± 1.64 | 64.11 ± 1.50 | 78.05 ± 3.11 |

Table S5. ROS scavenging ratio of Ce@P with different concentrations.

| Name | 50 μg/mL (%) | 100 μg/mL (%) | 200 μg/mL (%) |
| --- | --- | --- | --- |
| H_2_O_2_ | 23.21 ± 0.85 | 44.84 ± 1.46 | 59.82 ± 1.22 |
| ·OH | 25.49 ± 0.94 | 41.22 ± 0.89 | 45.71 ± 0.75 |
| ·O_2_^−^ | 34.22 ± 1.17 | 59.10 ± 8.96 | 76.20 ± 2.22 |

Table S6. Blood indicators of treated rats. The corresponding groups were: rats without treatment (sham group) and rats with Ce@P injection (Ce@P) after 7 day.

| Name | Sham | Ce@P |
| --- | --- | --- |
| PT (s) | 11.37 ± 0.25 | 11.50 ± 0.46 |
| INR | 1.03 ± 0.03 | 1.04 ± 0.04 |
| APTT (s) | 17.33 ± 0.67 | 17.57 ± 0.81 |
| TT (s) | 35.20 ± 3.21 | 35.53 ± 1.79 |
| FIB (g/L) | 1.59 ± 0.03 | 1.52 ± 0.05 |
| WBC (×10^9^/L) | 8.23 ± 0.40 | 7.13 ± 0.15 |
| RBC (×10^12^/L) | 7.81 ± 0.86 | 7.16 ± 0.75 |
| HGB (g/L) | 160.30 ± 17.62 | 145.70 ± 10.02 |
| PLT (×10^9^/L) | 1394.00 ± 349.50 | 1496.00 ± 165.50 |
| ALT (U/L) | 62.89 ± 11.22 | 75.50 ± 6.00 |
| AST (U/L) | 112.70 ± 9.35 | 119.70 ± 11.60 |
| CREA (μmol/L) | 48.29 ± 2.69 | 44.33 ± 5.00 |
| UREA (mg/dL) | 13.09 ± 2.70 | 11.90 ± 0.43 |
| CK (U/L) | 1079.00 ± 99.21 | 1100.00 ± 81.21 |
| CK-MB (U/L) | 326.50 ± 29.83 | 358.80 ± 43.54 |

Table S7. Blood indicators of treated rats. The corresponding groups were: rats without treatment (sham group), LPS induced rats with PBS injection (ALI group), LPS induced rats with Ce@P injection (Ce@P), and LPS induced rats with Ce@P injection and NIR irradiation (Ce@P + NIR).

| Name | Sham | ALI | Ce@P | Ce@P + NIR |
| --- | --- | --- | --- | --- |
| PT (s) | 10.10 ± 0.17 | 10.53 ± 0.40 | 10.17 ± 0.15 | 10.60 ± 0.10 |
| INR | 0.91 ± 0.02 | 0.95 ± 0.04 | 0.92 ± 0.02 | 0.96 ± 0.01 |
| PTA (%) | 117.00 ± 3.46 | 109.30 ± 7.10 | 115.70 ± 3.06 | 108.00 ± 2.00 |
| APTT (s) | 24.17 ± 1.89 | 20.87 ± 0.45 | 20.90 ± 1.51 | 26.10 ± 1.56 |
| WBC (×10^9^/L) | 5.89 ± 0.42 | 4.29 ± 1.04 | 4.41 ± 0.29 | 4.75 ± 0.96 |
| RBC (×10^12^/L) | 7.80 ± 0.38 | 7.76 ± 0.39 | 7.37 ± 0.58 | 7.67 ± 0.57 |
| HGB (g/L) | 144.70 ± 4.04 | 142.70 ± 11.37 | 137.00 ± 14.53 | 138.00 ± 9.00 |
| AST (U/L) | 115.70 ± 9.45 | 529.00 ± 123.40 | 410.70 ± 22.81 | 331.30 ± 30.86 |
| ALT (U/L) | 44.00 ± 8.19 | 110.30 ± 20.40 | 86.00 ± 19.47 | 74.67 ± 15.50 |
| UREA (mg/dL) | 6.50 ± 1.06 | 5.90 ± 0.41 | 5.97 ± 0.38 | 5.83 ± 1.12 |
| CREA (μmol/L) | 39.33 ± 7.02 | 32.00 ± 1.00 | 30.33 ± 9.29 | 33.67 ± 1.53 |
